# Quantized Constant-Q Gabor Atoms for Sparse Binary Representations of Cyber-Physical Signatures

**DOI:** 10.3390/e22090936

**Published:** 2020-08-26

**Authors:** Milton A. Garcés

**Affiliations:** 1 Infrasound Laboratory, University of Hawaii, Manoa, HI 96740, USA; milton@isla.hawaii.edu; 2 RedVox, Inc., Kailua-Kona, HI 96740, USA

**Keywords:** Gabor atoms, wavelet entropy, binary metrics, acoustics, quantum wavelet

## Abstract

Increased data acquisition by uncalibrated, heterogeneous digital sensor systems such as smartphones present new challenges. Binary metrics are proposed for the quantification of cyber-physical signal characteristics and features, and a standardized constant-Q variation of the Gabor atom is developed for use with wavelet transforms. Two different continuous wavelet transform (CWT) reconstruction formulas are presented and tested under different signal to noise ratio (SNR) conditions. A sparse superposition of Nth order Gabor atoms worked well against a synthetic blast transient using the wavelet entropy and an entropy-like parametrization of the SNR as the CWT coefficient-weighting functions. The proposed methods should be well suited for sparse feature extraction and dictionary-based machine learning across multiple sensor modalities.

## 1. Introduction

This paper applies the constant-Q standardized Infrasonic Energy, Nth Octave (Inferno) framework [1] to the Gabor wavelet [2] and proposes binary metrics for signature characterization. One of the primary motivations of this work is to facilitate the fusion of multi-modal data streams in sensor systems that collect information at different temporal and spatial granularities. Consider a cyber-physical sensor system that converts observables into digital time series data consisting of signals and noise. Signals of interest can be hypothetically described by sparse representations that define their signature. If the signature characteristics are sufficiently unique and recognizable from those of ambient coherent and incoherent noise, they can be used to identify and classify an object or process.

The transformation of diverse digital measurements into robust, scalable, and transportable representations is a prerequisite for signal detection, source localization, and machine learning applications for signature classification. The challenge at hand is to construct sparse signal representations that contain sufficient information for classification. Unambiguous classification can be elusive; measurement artifacts, unexpected signal variability, and non-stationary noise often conspire to add uncertainty to our classifiers. As will be discussed in this paper, information and uncertainty quantification can be substantially simplified when using standardized wavelets and binary metrics.

### 1.1. Binary Representations of Time and Frequency 

Oscillatory processes often exhibit spatial and temporal scalability and self-similarity. Although some physical processes scale linearly, many exhibit recurrent patterns that scale logarithmically and are well represented by power laws. Both linear and logarithmic scales can coexist. For example, overtones in harmonic acoustic systems are often linearly spaced in frequency, yet our sense of tone similarity is close to base 2 logarithmic (binary) octave scales. The term octave comes from the eight major notes in 12-tone musical notation, where every note frequency closely repeats with factors of two. This paper uses the term octave and binary interchangeably to denote the base 2 geometric scaling of frequency and time. The mapping between frequency (or pitch) and time (period) is direct for continuous tones, such as musical notes, or statistically stationary oscillations like the orbits of planets. Discrete Fourier transform methods are exceptionally well suited for the interpretation of steady tonal signals with linearly spaced harmonics. The Fourier transform deconstructs oscillations with distinct recurrent time periods into a *spectral* representation consisting of a set of discrete frequencies. The spectral transformation can be sparse because it removes time as a variable, facilitating the reconstruction of stable oscillations from a subset of coefficients in the Fourier spectrum.

Stable oscillators can be even more succinctly represented by a fundamental frequency *or* period (exclusive *or*, as they are not independent). For many physical systems, a map can be constructed between the fundamental frequency and its harmonics. Signals where the fundamental and its harmonics (when they exist) are statistically stationary and easily discernible above noise can be referred to as the easy continuous wave (CW) problem, or the zeroth (trivial) class of CW problems. The trivial CW problem is well understood and should routinely be used as a speed and performance benchmark for detection and classification algorithms.

The plot thickens when temporal variability is introduced in the signal or the noise. In the first class of CW problems, temporal variability is due to non-stationary broadband or band-limited noise. This is a chronic condition in infrasonic signal processing, where ambient noise can be coherent or incoherent across a dense sensor network [3] or an array aperture [4]. The first class of CW problems is also well understood when noise is predictable (e.g., normally distributed) over a time duration that is much longer or much shorter than the signal period in the detection band. However, this class of problems is not as well characterized when noise is not evenly distributed across the signal detection bandpass and can be particularly inconvenient when noise overwhelms the fundamental frequency band.

In the second class of CW problems, temporal variability is introduced by a change in the temporal, spectral, and/or statistical properties of the signal. These changes can be due to aging, failure, motion, communication, or any other change in state. In a simple two-state problem, one may quantify the properties of the first state, the transition period between states, and the properties on the final state. In a multiple-state problem, such as with communication systems, speech, or music, the Short-Time Fourier Transform (STFT) is often used to characterize spectral variability.

If the transition period between states is faster that the characteristic time scale of the initial state, the STFT does not always provide an accurate representation of this *transient*. For some signals, the details of the transient are not relevant and only the steady states are important. But a new class of signals emerges when the detection of transient anomalies is prioritized.

The zeroth class of transient problems consist of delta functions with their integrals and derivatives. Such instantaneous spikes do not exist in the natural world but can be readily constructed digitally to evaluate the impulse response of a system or represent a neuromorphic network [5,6]. The first class of transient problems would consist of realistic variants of the delta function that may be observed in the wild when a rapid change of state becomes the signal of interest. Just like a single-tone sinusoid may be regarded as the prototype end member for the trivial CW problem, an explosive detonation could be considered as a prototype transient signal source [7]. A time series corresponding to a blast would vary from ambient noise to a brief blast transient that fades back to a possibly perturbed background noise state. The transition from noise to signal can be devastatingly fast. In general, poorly-conditioned STFTs provides inadequate representations of brief, rapidly changing signals because the signatures no longer resemble a CW and are not optimally represented by sinusoids. However, since a STFT is a windowed sinusoid, a well-conditioned STFT window at the peak frequency of a signal turns the waveform in the STFT window into a wavelet that is well-tuned for the main signal bandpass.

The concept of a windowed sinusoid to represent a transient signal was introduced by Gabor [2] in 1946, and later mathematically formalized by others as wavelets. Variants of the Gabor wavelet are presented in the main text and the Appendix A, Appendix B, Appendix C, Appendix D, Appendix E, and Appendix F.

The second class of transient problems overlaps with the second class of CW problems. It corresponds to transients of significant durations which could be addressed with STFTs, wavelets, or their combination. Very often a transient is imbedded in a noise field with band-limited harmonic structure. Or the transient itself is a sweep, characterized by a substantial frequency change in the fundamental frequency and its harmonic structure.

The primary differences between STFTs and wavelet transform approaches are that the STFT uses a linear period mapping and a constant time window duration, while wavelets uses geometric pseudo-period mapping and time window durations that scales with the pseudo-period. Whereas in the Fourier framework there is a one-to-one mapping between time and frequency, the wavelet mapping between time scale and frequency can be less evident and depends on the selected wavelet.

This paper concentrates on developing highly standardized Gabor atoms [2] for the design and evaluation of transportable, sensor-agnostic transient signal detection, sparse feature extraction, and classification algorithms.

### 1.2. Binary Representations of Energy and Information in Cyber-Physical Systems

A Cyber-Physical System (CPS) is an algorithm-controlled computer system with physical inputs and outputs. A typical example of a mobile CPS is a smartphone with a microphone input (sound activation) that outputs a response (speech, music, or signal recognition) to a screen. Cyber-physical Measurement and Signature Intelligence (MASINT) is an emerging discipline that concentrates on phenomena transmitted through cyber-physical devices and their interconnected data networks. For smartphones and other multi-sensor mobile platforms connected to wireless networks, this includes digital noise, bit errors, and latencies internal to the device and its communication channels [8,9,10].

Data processed by the cyber part of CPSs are digital and represented as binary digits (bits). Although the precision of the data would be initially defined by its their allocated integer word size (16, 24 bit, etc.), the original data may be converted into floating point equivalents when an algorithms acts on them. For example, consider sound recorded by a smartphone at the standard rate of 48,000 samples per second. A typical sound record may have 16-bit resolution, so that its dynamic range in bits is 2^−15^ to 2^15^ – 1. However, one may only be interested in the lower frequency components of the raw data, so one would implement a lowpass anti-aliasing filter before decimation. Such filters often require floating point arithmetic in double precision (52 bit mantissa re IEEE 754 at the time of this writing) to reduce instability. Therefore, the precision of the resulting lowpass filtered data would exceed the specification of the original 16-bit integral input. However, the theoretical dynamic range of the system would not exceed the specification of the integer 16 physical input. Furthermore, data compression can be more efficient on floats than integers, which leads us to the topic of fractional bits as a measure of CPS amplitude, power, and information.

Many of the metrics we used in traditional physical and geophysical systems are inherited from the analog era. The base 10 decibel scale is a measure of power relative to a reference level, and is used extensively in telecommunications, acoustics, and electrical engineering. Let us estimate the hypothetical dynamic range of a 16-bit microphone record of a sinusoid at full scale. The peak rms amplitude would be

(1) $$p_{rms\;signal}=\frac{2^{16}}{2\sqrt{2}}\;.$$

All systems have quantization and system noise, and the noise can have a positive or negative bias. This is not a noise paper; for the sake of illustration, I model the system noise as oscillating around a mean of zero and alternating between −1 and 1,

(2) $$p_{rms\;noise}=\frac{2^{1}}{2\sqrt{2}}\;.$$

The theoretical dynamic range of the system in dB for a sinusoid recorded with a 16-bit microphone and sound card combination with a one-bit noise floor could be characterized by the ratio of the power

(3) $$10*log_{10}{\left[\frac{p_{rms\;signal}}{p_{rms\;noise}}\right]}^{2}=20*log_{10}\left[2^{15}\right]\approx 90\;\mathrm{dB}$$

where a digital response is converted to the legacy base 10 logarithmic system. One advantage of the decibel approach is that it can be compared to the response of the human ear and other analog systems. However, analog comparisons are not necessary for many cyber physical applications. A more natural unit for CPS is the binary logarithm

(4) $$log_{2}\left[\frac{p_{rms\;signal}}{p_{rms\;noise}}\right]=log_{2}\left[2^{15}\right]\approx 15.0\;\mathrm{fbits}$$

where the unit fbits corresponds to floating point representation of bits. For example, in 24-bit systems, present-day quantization error is ~3 bits, leading to an effective dynamic range of ~21 fbits. Likewise, a 24-bit integer cast into a 32-bit symbol can have 8 + 3 bits of noise, and may be converted to a float that still has ~21 fbits of dynamic range.

Another unit that is often specified is the ½ power point of the frequency response of a filter, which defines the quality factor of that filter. This is often referred to as the −3 dB point, since $10*log_{10}\left(2\right)\sim 3\;\mathrm{dB}$. However, accurate filter bank reproductions require a clear specification of the ½ power point, and conversion from base 10 to base 2 specification can lead to computational errors. Plotting filter responses in floating point bits can be informative as it reveals the precision of the computation. Because it is awkward and there is already a precedent in information theory for using bits outside of their original definition as a binary digit, from here onwards in this paper the word bits will be used to represent either the floating point equivalent of bits or as a metric for information.

Consider the communication channel capacity introduced by Shannon [11], which in its simplest form can be expressed as

(5) $$Ch=Wlog_{2}\left(\frac{Sg+Ns}{Ns}\right)$$

where Ch is a measure of the differential entropy of a signal in the presence of noise, *W* is a measure of the bandwidth, Sg is representative of the power of a signal, and *Ns* is representative of the noise power. The units of the channel capacity are in shannons per second, or bits per second, and represent the theoretical upper bound of the rate of information transfer in a communication channel. Since it is often impossible to separate noise embedded in a signal but it is often possible to construct a noise model, we can think the ratio *(Sg + Ns)/Ns* as a practical measure of the signal to noise ratio (SNR) of an observed signal that has been carried through a cyber-physical system or a medium.

The effective SNR and therefore the detectability of a compressed pulse (such as a wavelet) is the product of the bandwidth, the signal to noise ratio, and the time duration of a signal [12]. When using constant-Q Gabor wavelet with fractional octave (binary) bands n of order N and center frequency $f_{n}$ to process a signal in the presence of noise, the next section shows that for

(6) $$SNR_{n}=\frac{Ns_{n}+Sg_{n}}{Ns_{n}}=1+\frac{Sg_{n}}{Ns_{n}}$$

the signal detectability per band can be represented by

(7) $$bSNR_{n}=\frac{1}{2}\;log_{2}\left(SNR_{n}\right)$$

and the upper limit on rate of information in bits per second for a band-limited pulse with center frequency $f_{n}$ can be estimated from

(8) $$Ch_{n}=\;\frac{f_{n}}{N}\;bSNR_{n}\;.$$

Energy and Shannon entropies using the binary log are constructed for both the wavelet coefficients and SNR in Section 2.5.

## 2. Methods

This is an algorithmic paper providing foundational methods to construct standardized Gabor wavelets within a binary framework. No materials are included or required; all the algorithms required to reproduce the results are presented, with recommendations for specific existing functions in open-source software frameworks.

Although the methods are intended to be sensor-agnostic and transportable across diverse domains, the selection of the Gabor mother wavelet does define the optimal applicability of the algorithms: the methods in this paper will work best with a transient, or a portion of a transient, that can be well represented by a superposition of Gabor wavelets. Fortunately, this covers a fairly wide range of transient signature types. The fundamental principles in this work are expandable to other wavelets as well as to four-dimensional spatiotemporal representations.

### 2.1. Transforming Time and Frequency to Scale

A digital time series is constructed by collecting digital measurements at discrete times separated by a nominal sample interval $\Delta \tau_{s}$. One may estimate a standard deviation from nominal $\sigma_{\tau_{s}}$ associated with the sample interval; when this error is a very small percent of the sample interval (e.g., parts per million) it is generally treated as a constant. Some variability in the sample rate should be expected in cyber-physical sensing systems under different conditions (temperature, battery level, power load, data throughput, etc.) even when the systems have the same hardware configurations. This can have an impact when attempting high-accuracy time synchronization. If adequate performance metrics are collected, the sample rate error can be quantified and potentially compensated by an additional time-varying correction to the clock drift.

In many scientific domains, such as astronomy and climatology, the sample interval may be greater than one second. Domains where the phenomena of interest change more rapidly use the equivalent metric of samples per second, referred to as the sample rate and often expressed in units of Hertz. The relationship between the sample interval $\Delta \tau_{s}$ and its standard deviation $\sigma_{\tau_{s}}$ and the sample rate $f_{s}$ and its associated error can be expressed as

(9) $$\frac{1}{\Delta \tau_{s}+\sigma_{\tau_{s}}}=\frac{1}{\Delta \tau_{s}}{\left(1+\frac{\sigma_{\tau_{s}}}{\Delta \tau_{s}}\right)}^{-1}\approx \;f_{s}\left(1-\frac{\sigma_{\tau_{s}}}{\Delta \tau_{s}}\right)\;\mathrm{if}\;\frac{\sigma_{\tau_{s}}}{\Delta \tau_{s}}\ll 1\;.$$

Although time is the primary discrete sampling parameter, system requirements are often provided as frequency specifications within the context of Fourier transforms. The nominal sample rate sets the maximum upper edge of the bandpass of the system; there should be negligible energy at the Nyquist frequency, which is half of the sample rate. The actual bandpass of a system is set by the low- and high- frequency cutoffs of a cyber-physical system, which may include the sensor response, hardware specifications, firmware and software modifications (such as anti-aliasing filtering), and data compression.

The mapping between frequency and period is simple for a continuous wave tone; the tone period is the inverse of the tone frequency. It is not so clear for transients. Following [7], a transient with a single spectral peak at a center frequency $f_{n}$ may be associated with a pseudo-period $\tau_{n}=1/f_{n}$. This mapping is important as the scale of wavelet representations is linearly proportional to the pseudo-period, which is also referred to as the scale period. A high-level overview of the Appendix A, Appendix B, Appendix C, Appendix D, Appendix E, and Appendix F is provided in this section for ease of reference.

Constant quality factor (Q) bands with constant proportional bandwidth are traditionally defined as [1]

(10) $$\frac{\Delta f}{f_{n}}=\frac{1}{Q}$$

where Δf is the bandwidth centered on $f_{n}$. The Q is a measure of the number of cycles needed to reach the ½ power point at the bandwidth edges. Appendix A shows that the bandwidth edges are well defined in fractional octave band representations of order N so that the quality factor can be evaluated precisely as,

(11) $$Q_{N}={\left[\;2^{\frac{1}{2N}}\;-\;2^{-\frac{1}{2N}}\right]}^{-1}.$$

From [1], and as shown in Appendix B and Appendix C, the characteristic time duration of the Gabor atom can be represented as

(12) $$T_{n}=M_{N}\;\tau_{n}$$

where $M_{N}$ is a measure of the number of oscillations in the characteristic time duration of a wavelet. For efficient computation all physical times are nondimensionalized and converted to equivalent sample points by multiplying by the sample rate. If t is the time in seconds, the nondimensionalized time m is

(13) $$m=f_{s\;}t\;.$$

The approach is wavelet-agnostic up to this stage. Direct application of the ½ power points of the spectrum of Gabor-Morlet wavelet (Appendix B) at the band edges (Appendix C) yields

(14) $$M_{N}=2\sqrt{ln2}\;Q_{N}\approx 2\sqrt{2ln2}\;N$$

where $M_{N}$ controls the duration of the wavelet to match the order’s *Q*. This last step can be tailored to other wavelet types to produce constant-Q variants. Adherence to the specifications in Equations (10)–(14) yield standardized and well-constrained quantized Gabor atoms. 

### 2.2. Binary Quantized Constant-Q Gabor Atoms

Gabor [2] extended the Heisenberg principle to define the time-frequency uncertainty principle, and further proposed deconstructing signals into elementary waveforms referred to as time-frequency atoms [2,13]. These atoms provide the optimum compromise between time and frequency resolution and thus maximize information density. The Morlet wavelet [14,15], functional kin to the Gabor atom, was developed for seismic applications and is much beloved by mathematicians. Much has been said and written over the last 75 years about the merits, and limitations, e.g., [16], of the Gabor atom in diverse fields of applied science ranging including quantum mechanics, e.g., [17], neurophysiology, e.g., [18] and radar target recognition, e.g., [19]. 

Consider the translation and dilation of the familiar Gabor-Morlet mother wavelet

(15) ΨN(m)=1π14exp(−m22) exp(iMNm)

with dictionary [13]

(16) $$\Psi_{n}\left[m-m^{\prime }\right]=\frac{1}{\sqrt{{𝓈}_{n}}}\Psi_{N}\left(\frac{m-m^{\prime }}{{𝓈}_{n}}\right)$$

which can be fully expressed as

(17) Ψn(m−m′)=1π141𝓈nexp{−12[m−m′𝓈n]2}exp{iMN[m−m′𝓈n]}

where the mapping between the nondimensional scale ${𝓈}_{n}$ and the band period is

(18) $${𝓈}_{n}=\;\frac{M_{N}}{2\pi }\;f_{s}\tau_{n}\;.$$

The constant-Q Gabor atoms are constrained to the discrete set of values

(19) $${𝓈}_{n}=\;{𝓈}_{0}2^{\frac{n}{N}}=\frac{M_{N}}{2\pi }\;f_{s}\tau_{0}2^{\frac{n}{N}},M_{N}=2\sqrt{ln2}\;Q_{N}$$

with quality factor

(20) $$Q_{N}={\left[\;2^{\frac{1}{2N}}\;-\;2^{-\frac{1}{2N}}\right]}^{-1}\approx \sqrt{2}N$$

defined by the ½ power points of the Fourier spectrum, quantized order N. For this functional form, the wavelet admissibility condition can be represented as

(21) $$M_{N}^{2}\;\gg 1\;.$$

By quantizing constant-Q bands and the resulting wavelet scales it is possible to also discretize the uncertainty in time and frequency of the resulting analyses. Gaussian pulses in general [12] and Gabor atoms in particular are well-known to have the lowest time-frequency uncertainty [2,13], making them natural building blocks for uncertainty quantification. The Gabor atom has the minimal value of the Heisenberg-Gabor uncertainty (Appendix D), where the nondimensionalized temporal standard deviation $\sigma_{t}$ and angular frequency standard deviation $\sigma_{\omega }$ over all time and frequency satisfy

(22) $$\sigma_{f_{s}t}\;=\;\frac{1}{\sqrt{2}}{𝓈}_{n}\;\Rightarrow \sigma_{t_{n}}\;=\;\frac{1}{\sqrt{2}}\frac{M_{N}}{2\pi }\tau_{n}$$

(23) $$\sigma_{\omega /f_{s}}=\;\frac{1}{\sqrt{2}}{𝓈}_{n}^{-1}$$

(24) $$\sigma_{t}\sigma_{\omega }=\;\frac{1}{2}$$

which quantify time and frequency uncertainty discretely, minimally, and unambiguously.

Converting to physical time with $m=f_{s\;}t$ yields a more familiar Morlet representation

(25) Ψn(t−t′)=1(π𝓈n2)14exp{−12[ fs(t−t′)𝓈n]2}exp{i2πfn fs[ fs(t−t′)]}

where the scale ${𝓈}_{n}$ may be readily recognized as the standard deviation of a Gaussian envelope with integration variable $m=\;f_{s}t$. This is very similar to the original form proposed by Gabor [2], and makes intuitive sense as the oscillatory term is clearly exposed. However, the additional factor of $f_{s}$ required to nondimensionalize the numerator of the Gaussian envelope for numerical computation has indubitably been an initial source of confusion amongst some physical scientists, author included.

### 2.3. Quantum Order

The recommended quanta for the Gabor atoms are positive integer band numbers n and the preferred orders N as in [1]

(26) n=0, 1, 2…,   N=1, 3, 6, 12, 24…

though the special orders *N* = 0.75 and 1.5 are considered. The mother wavelet is uniquely defined (and can be quantized) by the order *N*, although it is often specified by the more accessible variable $M_{N}$. The mother wavelet is scale invariant. Each discrete atom in its dictionary is defined by its order *N*, its band number n, and a refence scale at *n* = 0. If the Gabor atoms remain within their quanta, there is only one degree of freedom: the reference scale. The reference scale can be set by the data acquisition system (e.g., the Nyquist frequency) or a standard reference frequency. The scale schema can also be set by a signal tuning frequency; the theoretical peak acoustic frequency for the detonation of one metric ton of TNT is used in Section 3. When integrating multi-sensor time series with different evenly and unevenly sampled data, it would be preferrable to either use a standard reference frequency or time scale (e.g., 1 kHz for audio, 1 Hz for infrasound [1]) or a shared target frequency. The resulting frequency bands will be evenly spaced logarithmically to standardize and facilitate multi-sensor cross-correlations and data fusion. It is important to reinforce that the mapping from physical time scale to nondimensional scale depends on the sample rate. Specifying a nominal sample rate $\;f_{s}$ or sample interval $\Delta \tau_{s}=1/f_{s}$ as in Equation (9) permits conversion to physical time t and scale pseudo-period $\tau_{n}$ from the wavelet parameters,

(27) $$t=\frac{m}{\;f_{s}},\tau_{n}=\frac{2\pi }{M_{N}}\frac{{𝓈}_{n}}{\;f_{s}},$$

and map to the physical scale center frequencies

(28) $$f_{n}=\frac{1}{\tau_{n}},\;\omega_{n}=2\pi f_{n}\;.$$

It may be useful to think of the binary (base 2) order *N* as the quantized time and bandwidth stretch factor of the Gabor atom; as the order increases, the wavelet stretches in time and narrows in bandwidth, with each frequency band occupying a constant proportional frequency bandwidth that produces $Q_{N}$ oscillations at the band frequency in the time domain. Although in theory it is possible to use any integer band indexes *n*, for computational implementation it is practical to use only nonnegative integers to represent temporal scales [Equation (26)], with $\tau_{0}$ corresponding to the smallest scale and $f_{0}$ to the highest center frequency below the Nyquist frequency. 

This paper recommends atom quantization using the well-established fixed order N and quality factor $Q_{N}$ values of standard geometric binary intervals referred to as fractional octave bands in acoustic and infrasound applications (Table 1).

Appendix A develops a useful approximation for the quality factor $Q_{N}$ of order *N*,

(29) $$Q_{N}\approx \sqrt{2}N\approx 1.414\;N,M_{N}=2\sqrt{ln2}\;Q_{N}\approx 2\sqrt{2ln2}\;N\approx \;2.355\;N$$

with exact equivalence for octave bands at *N* = 1 (Table 2).

These relations are seldom made explicit for constant-Q wavelet representations, which often leads to inadvertently creative interpretations and implementations. In traditional fractional octave bands, N is an integer with preferred numbers 1, 3, 6, 12, 24 and its half-power (−3 dB) band edges and center frequencies are well established so their *Q* can be readily computed (Table 1 and Table 2). The band spectrum will overlap at the half-power point band edges to reduce (or at least regulate) spectral leakage and improve energy estimation. Dyadic wavelets use order *N* = 1 and are weakly admissible ($\;M_{N}^{2}\sim 5.54$); carefully handled they do lead to very sparse and fast computational implementations (e.g., [13]). 

The estimate for $Q_{N}$ in terms of the order N is useful for practical application where we wish to specify the number of oscillations $Q_{N}$ in a window. If one abandons the bounds of the preferred bands, one can estimate the order for a wavelet that has any number of oscillations in its support window. Once N is estimated, exact values for the center frequencies and band edges can be computed from the expressions in Appendix A. These bespoke constant-Q bands will not meet binary (factor of two) center frequency recursions with ½ power band edge overlap, but may be useful for highly customized tuning. Examples are provided in Table 3.

Consider the curious case of a single oscillation in the window, where

(30) $$N=\frac{3}{4}=0.75,\;Q_{0.75}=1.04,\;M_{0.75}=2\sqrt{ln2}\;Q_{0.75}\approx 1.73$$

and *Q* is evaluated more precisely from the order *N*. Although intuitive and compact, the resulting wavelets are marginally admissible ($\;M_{0.75}^{2}\sim 3\;$) and produce oddly spaced, but legitimate, constant-Q frequency bands that grow rapidly and hit only every fourth standard octave every three bands. The window duration will be only 1.74 periods long and the spectral resolution of the Fourier transform will be exceedingly sparse. Adding another oscillation per window (increasing the quality factor to approximately two), would correspond to

(31) $$N=\frac{3}{2}=1.5,\;Q_{1.5}=2.14,\;M_{1.5}=2\sqrt{ln2}\;Q_{1.5}\approx 3.56.$$

The resulting wavelets that are more admissible ($\;M_{1.5}^{2}\sim 13\;$) but also produce oddly spaced constant-Q frequency bands that land on every second standard octave every three bands. Third order bands hit exact powers of two every third band and have around four oscillations per window (Appendix C). Although it is possible to force center frequency scales, if best practices for band overlap are ignored one will have a set of wavelet filter banks with substantial spectral leakage or gaps between adjacent bands, and the possibility for excessively overdetermined or underdetermined results. This is what usually happens with default parameters on most continuous or discrete wavelet transform algorithms. This paper standardizes and regulates band spacing by asserting the relationship between order, bandwidth, and duration. Since it is both silly and mathematically inadvisable (even inadmissible) to construct a wavelet with less than one oscillation in its window, it is recommended that Q≥1. This suggests a minimum order number (quantum) of *N* = 3/4 for stable Gabor atoms, with *N* = 1 yielding value exact power of two (binary) bands.

It is possible to estimate the smallest possible universal binary scale from the Planck time, the smallest measurable time scale

(32) $$\Delta \tau_{Planck}=10^{-43}s\sim 2^{-142}s\;.$$

Since the Planck time would be the smallest possible sample interval, the smallest oscillation that could be observed would be at the universal Nyquist period

(33) $$\tau_{min}=2\Delta \tau_{Planck}\sim 2^{-141}s\;.$$

At the other end of the timeline, the age of the universe is estimated to be 13.8 billion years, or

(34) $$\tau_{max}\sim 2^{58}\;s,$$

so that all time scales in the known universe can be encompassed within ~200 temporal octave bands. Computationally speaking, this is a small range of octaves that can be spanned by 200 Gabor atoms. Earth is estimated to be ~4.6 billion years old, covering around about 57 of those temporal binary bands. The oldest bones associated with Homo Sapiens-Sapiens are ~200,000 years old and within the last 42 temporal sub-bands since Earth’s inception. The human voice for average individuals ranges between one and two octaves, and five octaves species-wide. The nondimensionalized scale ${𝓈}_{Nyquist}$ of the binary (*N* = 1) Gabor atom at the Nyquist frequency is always the same whether one uses the Plank scale or a sample rate of 48 kHz

(35) $$\;Q_{1}=\sqrt{2},\;M_{1}=2\sqrt{2ln2}$$

(36) $${𝓈}_{Nyquist}=\frac{M_{1}}{2\pi }\frac{f_{s}}{f_{Nyquist}}=\frac{M_{1}}{2\pi }\frac{\tau_{min}}{\Delta \tau_{Planck}}=\;\frac{2\sqrt{2ln2}}{\pi }\;\approx 0.75.$$

However, it is inadvisable make observations at the Nyquist limit, and it would be preferable to consider the starting center scale at one quarter of the sample rate, or four times the sample period. It would be possible to construct universal time scales with $\tau_{0}=\;2^{-140}s$, whereas all timescales would occupy temporal sub-bands. The corresponding sensor-agnostic nondimensionalized scale would be $2\;{𝓈}_{Nyquist}$.

A third order representation (*N* = 3) of all the times scales in the universe can be represented by 600 temporal Gabor atoms. The beauty of the third order representation is that it is very close to the decimal representation, with every ten 1/3 octaves producing a decade $\left(2^{10/3}\sim 10\right)$, and thus provide a geometrically elegant compromise between ten-digit humans and binary digit machines. In addition to better meeting the admissibility condition, third order bands will contain over 99% of the information within their octave (Appendix D), making them compact temporal carriers. If the third order representation is used as the base order (*N* = 3), the preferred numbers are binary multiples (*N* = 3, 6, 12, 24 in Table 1), with a proportional elongation in the wavelet support and increase in spectral resolution.

Many software packages readily produce a Gabor-Morlet wavelet with default parameters (Appendix E). One of the most common values is $M_{N}=5$, which is close to order N=2 (Table 4). Other common values of the wavelet support correspond to $M_{N}=4,\;N=1.7$ and the more reasonable $M_{N}=8$ which is close to preferred order N=3.

Because none of these specifications correspond to standard orders, the resulting wavelets will tend to either overestimate (due to spectral leakage) or underestimate (due to spectral gaps between bands) the energy within adjacent constant-Q bands if binary center frequencies are forced, or will produce non-standard center frequencies.

Although it is possible to quantize the constant-Q Gabor atoms using the order *N*, the quality factor *Q*, or the multiplier $M_{N}$, the order is the most logical way to define the quanta of the wavelet. Describing the proposed wavelet dictionaries of preferred orders as the quantized constant-Q Gabor atoms with binary bases and overlapping ½ power points is rather awkward, and this paper proposes referring to these constructs as quantized wavelets, quantum wavelets of order *N*, or Nth order Gabor atoms. Although *N* = 1 provides a sparse clean binary (with power of two steps in frequency) representation with the tightest windows, the admissibility condition coupled with the better reconstruction capability presented in the next section suggests that using *N* = 3 as the base order is preferable, with the added advantage that all subsequent preferred orders in Table 1 are binary factors of base order 3.

### 2.4. Continuous Wavelet Transform Deconstruction and Reconstruction

The continuous wavelet transform (CWT) of a function g(x) is represented in [13] (Equation (1.13)) as

(37) $$W\left(g,\;u,𝓈\;\right)=g,\Psi_{u,n}=\int_{-\infty }^{\infty }g\left(x\right)\frac{1}{\sqrt{𝓈}}\Psi^{*}\left(\frac{x-u}{𝓈}\right)dx$$

where the asterisk (*) represents the complex conjugate. The equivalent CWT for a discrete sequence of observations (or a synthetic time series) g(m) is the convolution of g with a scaled and translated version of Ψ(m). Consider the nondimensional Quantum mother wavelet of order *N*,

(38) ΨN(m)=1π14exp(−m22) exp(iMNm)

(39) $$\Psi_{n}\left[m\right]=\frac{1}{\sqrt{{𝓈}_{n}}}\Psi_{N}\left(\frac{m}{{𝓈}_{n}}\right)\;.$$

The discrete CWT can be expressed as

(40) $$W_{n}\left[m\right]=\overset{Mp-1}{\underset{m^{\prime }=0}{\sum }}g\left(m^{\prime }\right)\Psi_{n}^{*}\left(m^{\prime }-m\right)=g⊛\Psi_{n}^{*}\left[m\right]$$

where the symbol ⊛ denotes a convolution [13], often computed using the discrete Fourier transform. This is comparable to the expression in [20], although their convolution has no amplitude scaling as it is corrected afterwards. The CWT coefficients $W_{m,n}$ provide a measure of the degree of similarity between the time series and the wavelet of scale index *n* while translating along the time index *m*. While exact waveform reconstruction from the CWT is challenging (e.g., [21,22]), reference [20] provides an approximate expression for the wavelet-filtered time series $g\left(m^{\prime }\right)$. The reconstruction filter from the Nth order Gabor atoms becomes,

(41) $$g\left[m\right]\approx \frac{\pi^{\frac{1}{4}}}{N}\frac{1}{C_{\delta }}\overset{Np-1}{\underset{n=0}{\sum }}\frac{Re\left\{W_{n}\left[m\right]\right\}}{\sqrt{{𝓈}_{n}}}$$

where *Re*{ } denotes the real part of the coefficients and the reconstruction factor $C_{\delta }$ is scale independent and constant for wavelet function with fixed $M_{N}$. The reconstruction factor can be estimated by comparing against known test functions. Reference [20] empirically computed a reconstruction coefficient of $C_{\delta }=0.776$ with $M_{N}=6$, and [23] provides other estimates. Numerical evaluation shows the product $NC_{\delta }\sim 2$, and the reconstruction approximation for the analytic (Appendix F) quantum wavelet of arbitrary order is

(42) $$g_{\mathbb{C}}\left[m\right]\approx \frac{\pi^{\frac{1}{4}}}{2}\;\sum_{n=0}^{Np-1}\frac{W_{n}\left[m\right]}{\sqrt{{𝓈}_{n}}}\;.$$

It is important to note how substantially different this expression is to the inverse discrete Fourier transform, where

(43) $$g_{DFT}\left[m\right]=\frac{1}{\sqrt{Np}}\;\overset{Np-1}{\underset{n=0}{\sum }}{\hat{g}}_{DFT}\left[n\right]exp\left(j2\pi mn/Np\right)$$

and ${\hat{g}}_{DFT}\left[n\right]$ are the Fourier coefficients. Unlike the discrete Fourier transform, the standard wavelet reconstruction does not require multiplication by the mother wavelet. For the special case where the atoms are well matched to the signal of interest, consider the sparse set of coefficients corresponding the complex time indexes $m_{n\;\mathbb{C}\;max}$ of the maximum energy, entropy, or SNR at each scale

(44) $$g_{\mathbb{C}\;}\left[m\right]\approx \frac{\pi^{\frac{1}{4}}}{2}\;\overset{Np-1}{\underset{n=0}{\sum }}\frac{W_{n}\left[m_{n\;\mathbb{C}\;max}\right]}{\sqrt{{𝓈}_{n}}}Re\left\{\Psi_{n}\left[m-m_{n\;\mathbb{C}\;max}\right]\right\}$$

where the maximum coefficient indexes can be computed separately for real and imaginary components. This has the form of a sum over the dominant Gabor atoms for each scale. Since one is only considering the maxima in a given record window, this is a very sparse representation consisting of the coefficient and the time offset corresponding to the peak energy or entropy estimate. Numerical evaluation shows that this last expression can be used to estimate the full analytic function representation as long as reconstruction uses the complex coefficients but only the real atom function since the time shifts in the Hilbert transform already include the π/2 time shift.

### 2.5. Wavelet Information and Entropy

One advantage of the constant Q wavelet representation is that it is possible to estimate the information content and detectability of a signal in a band by applying the same set of wavelet transforms to the signal and comparing them to the transform of a noise segment or model. Consider the definition for Shannon’s channel capacity [11], with

(45) $$SNR_{n}=\frac{Ns_{n}+Sg_{n}}{Ns_{n}}=1+\frac{Sg_{n}}{Ns_{n}}$$

(46) $$Ch_{n}=\;Wlog_{2}\left(SNR_{n}\right)$$

where *Sg* is the wavelet-transformed signal power and *Ns* is the wavelet-transformed noise power in a band. Consider two possible estimates for the bandwidth *W* (Shannon [11] left some room for interpretation). The first estimate approximates *W* by the ½ power point bandwidth

(47) $$\Delta f_{n}=\frac{f_{n}}{Q_{N}}\approx \frac{1}{\sqrt{2}}\frac{f_{n}}{N}\approx 0.7071\;\frac{f_{n}}{N}\;.$$

The second estimates *W* using the Gabor box standard deviation for the angular frequency

(48) $$\sigma_{\omega }=\frac{1}{\sqrt{2}}\frac{\omega_{n}}{M_{N}}\approx \frac{1}{4\sqrt{ln2}\;}\frac{\omega_{n}}{N}\approx \;\frac{\pi }{2\sqrt{ln2}\;}\frac{f_{n}}{N}\approx 1.8867\;\frac{f_{n}}{N}$$

so that

(49) $$\sigma_{f}=\frac{\sigma_{\omega }}{2\pi }\;=\frac{1}{4\sqrt{ln2}\;}\frac{f_{n}}{N}\approx 0.3003\;\frac{f_{n}}{N}\;.$$

Taking the average of $\Delta f_{n}$ and $\sigma_{f}$ provides a compromise between the two possible estimates, and a returns a tidy factor of ~0.5

(50) $$Ch_{n}\approx \;\frac{1}{2}\frac{f_{n}}{N}log_{2}\left(SNR_{n}\right)\;.$$

The effective $SNR_{G}$ and therefore the “detectability” of a bandwidth-limited compressed pulse [12] can be represented by the product of the Gabor time-bandwidth product (Appendix C) and the signal to noise ratio

(51) $$\;SNR_{G}=\sigma_{t}\;\sigma_{\omega }\times SNR_{n}\;.$$

Since the time-bandwidth product for the Gaussian wavelet is constant

(52) $$\sigma_{t}\sigma_{w}=\frac{1}{2}$$

and the uncertainty of its Gabor box is at the minimum, the likelihood of the detection of a signal of interest in a given band n is only proportional to its SNR.

Shannon’s definition of the channel capacity was intended to represent the highest theoretical transfer rate of information through an analog line. Since SNR is given in power, which is typically the square of the signal amplitude, an unscaled binary log is off by a factor of two from the original data in bits. To reconcile this definition with the original collection of a time series signal in floating point bits (fbits), I define the binary SNR to match the signal rms amplitude as well as Shannon’s units for the information rate per band $Ch_{N,n}$ of the quantum compressed pulse as

(53) $$bSNR_{n}=\frac{1}{2}log_{2}\left(SNR_{n}\right)=log_{2}\left(\sqrt{SNR_{n}}\right),\;fbits$$

(54) $$Ch_{N,n}=\frac{f_{n}}{N}\times bSNR_{n},\;shannons/s=fbits/s.$$

The increase in higher information delivery rate with increasing frequency is intuitive as more cycles are transferred per second. As the order number increases, the bandwidth narrows and so the potential information rate decreases. Less obvious is the decrease in high-frequency information with increasing distance in a lossy transmission channel. Assuming the noise power remains unchanged, the decrease in SNR with increasing scaled distance r from the source origin on a lossy acoustic channel can be represented as

(55) $$SNR=SNR_{o}\;\frac{exp\left(-\gamma f^{2}r\right)}{r^{n_{g}}}.$$

where $n_{g}=2$ for spherical geometric spreading in free space and $n_{g}=1$ for cylindrical spreading in a waveguide. The binary SNR can be represented as

(56) $$SNR=\left[bSNR_{0}-\frac{n_{g}}{2}log_{2}r\;\right]\;-\;f^{2}r\;\left(\gamma \;log_{2}e\right).\;$$

The term in parenthesis shows the expected reduction of one bit per doubling of distance for spherical spreading $\left(n_{g}=2\right)$. The last term suggests the frequency dependence of the channel capacity in a lossy acoustic medium may have the general form

(57) $$Ch_{n}\sim \alpha \left(log_{2}r\right)\;f-\beta \left(r\right)\;f^{3}$$

so that with increasing range the optimal information transmission frequency shifts to lower frequencies. One may readily extend the binary SNR definition to the measure of relative power

(58) $$bR=log_{2}\left(\sqrt{\frac{S}{S_{max}}}\right)=\frac{1}{2}log_{2}\left(\frac{S}{S_{max}}\right),fbits$$

and the −3dB half-power point becomes the −1/2 bit power point.

The entropy of a signal of interest can be estimated by the wavelet coefficients. A practical approach is described in [24]. The information content of each scale *n* at the time step *m* can be estimated from the wavelet energy. First estimate the complex wavelet coefficient energy from

(59) $$E_{m,\;n}={\left|Re\left\{\;W_{m,n}\right\}\right|}^{2}+j\;{\left|Im\left\{\;W_{m,n}\right\}\right|}^{2}\;.$$

The total energy in a given record can be estimated from

(60) $$E=\;\underset{m}{\sum }\underset{n}{\sum }\sqrt{E_{m,\;n}E_{m,n}^{*}}\;.$$

The complex probability of $W_{m,n}$ in the record is

(61) $$p_{m,n}=\frac{E_{m,\;n}}{E}$$

where

(62) $$\underset{m}{\sum }\underset{n}{\sum }p_{m,n}p_{m,n}^{*}=1$$

The log energy entropy (lee) per coefficient can be defined by the binary logarithm

(63) $$e_{lee}=log_{2}\left(p_{m,n}^{2}\right)=2log_{2}\left(p_{m,n}\right)$$

where it should be noted that the factor of two scaling coefficient does not alter the relative weight of each coefficient. The Shannon entropy (se) per CWT coefficient is defined as

(64) $$e_{se}=-p_{m,n}log_{2}\left(p_{m,n}\right)$$

with corresponding complex versions that separate the real and imaginary components. These entropies can be readily evaluated to construct noise models from the lowest entropy components. If a stable noise model can be constructed from the record or from prior knowledge of the environment and transmission channel, SNR estimates can be computed and the process repeated to evaluate the dimensionless binary log of the SNR

(65) $$bSNR_{m,n}=\frac{1}{2}log_{2}\left(SNR_{m,n}\right)$$

and the product of the ratio and the binary ratio (*RbR*), an entropy-like nondimensional metric of the SNR that can be readily evaluated to identify and extract the wavelet coefficients would be most representative of a signal of interest,

(66) $$RbR_{m,n}=\;SNR_{m,n}\times bSNR_{m,n}\;.$$

## 3. Discussion: Explosion Signature

The methods presented in this paper are foundational: the intention is to use the Gabor atoms as fundamental building blocks with minimal time-frequency uncertainty and high information density. These methods are illustrated and discussed in the context of a blast pressure pulse. Consider a normalized transient wave function characteristic of an explosion. Suppose one wanted to construct a sparse wavelet representation of a blast pulse with peak energy at 6.3 Hz, corresponding to the detonation of one metric ton of TNT observed at 1 km. It is known [7] that at some distance from the source this center frequency may drop by an octave (factor of two in frequency) or more, as well as become stretched out (dispersed) in time due to propagation effects. A theoretical source pressure function for the detonation of high explosives was developed in some detail in [7] with one kiloton as the case study, and is used here to construct a representative synthetic waveform for a one (metric) ton detonation. Define $\tau_{c}$

(67) $$\tau_{c}=4\tau_{p},f_{c}=\frac{1}{\tau_{c}},\;\omega_{c}=2\pi f_{c}$$

as the pseudo-period of a blast pulse corresponding to the peak spectral energy at the frequency $f_{c}$ and angular frequency $\omega_{c}$, where $\tau_{p}$ is the time duration of the initial positive phase traditionally used in blast physics. The nondimensionalized time scale is

(68) $$\hat{\tau }=\frac{t}{\tau_{p}}=4\frac{t}{\tau_{c}}\;.$$

The form of the amplitude-normalized source pressure function for an explosive blast [7] can be represented as

(69) $$g\left(\hat{\tau }\right)=\left(1-\hat{\tau }\right),\;0\leq \hat{\tau }\leq 1$$

(70) $$g\left(\hat{\tau }\right)=\frac{1}{6}\left(1-\hat{\tau }\right){\left(1+\sqrt{6}-\hat{\tau }\right)}^{2},\;1<\hat{\tau }\leq 1+\sqrt{6}\;.\;$$

This pulse has an associated analytic function $g_{\mathbb{C}}\left(\hat{\tau }\right)$ discussed in Appendix F. Since the theoretical Hilbert transform has some unresolved issues, the numerical Hilbert transform [25] is used for comparison.

Note that the amplitude is not used in this exercise because in some cyber-physical systems, such as smartphones, the amplitude response of on-board sensors may not be known. However, sensor dynamic range is usually specified and available (e.g., int16, float32) and can be used for signal scaling relative to the full range or the noise.

The normalized pulse has zero mean (conservation of momentum) and its theoretical variance is

(71) $$\sigma_{p}^{2}=\int_{-\infty }^{\infty }g^{2}\left(\hat{\tau }\right)dt=0.95\frac{\tau_{c}}{8}$$

The complex Fourier transform $\hat{g}\left(j\hat{\omega }\right)$ of this pulse is

(72) $$\hat{g}\left(j\hat{\omega }\right)=\frac{\pi }{2\omega_{n}\;}\left[\frac{1-j\hat{\omega }-e^{-j\hat{\omega }}}{{\hat{\omega }}^{2}}+\;\frac{e^{-j\hat{\omega }\left(1+\sqrt{6}\right)}}{3{\hat{\omega }}^{4}}\left\{j\hat{\omega }\sqrt{6}+3+\;e^{j\hat{\omega }\sqrt{6}}\left[3{\hat{\omega }}^{2}+j\hat{\omega }2\sqrt{6}-3\right]\right\}\right]$$

where $\hat{\omega }=\frac{\pi }{2}\frac{\omega }{\omega_{c}}=\frac{\tau_{c}}{4}\omega =$ $\tau_{p}\omega$ and the peak in the spectrum is at $\omega =\omega_{c}$. Note there are at least two pseudoperiods of importance evident in the main blast pulse: the main spectral pseudoperiod $\tau_{c}$ and the positive phase pseudoperiod of $2\tau_{p}$. Near the source the positive phase pseudoperiod will dominate as it has the highest energy and bandwidth. With increasing distance and high-frequency attenuation the main pseudoperiod becomes more prominent and may also be downshifted in frequency [7]. However, additional scales can be introduced by reflection and refraction in the transmission channel that can induce phase shifts often modeled with Hilbert transforms (Appendix F).

The power spectra of real digital signals are usually expressed using only the positive frequencies up to the Nyquist frequency, where the unilateral spectral density $Pg\left(\hat{\omega }\right)$ is defined as

(73) $$Pg\left(\hat{\omega }\right)=2{\left|\hat{g}\left(j\hat{\omega }\right)\right|}^{2}=2\;\hat{g}\left(j\hat{\omega }\right){\hat{g}}^{*}\left(j\hat{\omega }\right).\;$$

Since the target signature corresponds to a one tonne (1000 kg) detonation, the analysis concentrates on a target frequency of 6.3 Hz [7]. The general procedure for constructing target-tuned fractional binary bands of order N is to define a set of base 2 scales around the center or reference frequency

(74) $$f_{c}=6.3\;\mathrm{Hz},\;f_{j}=f_{c}2^{\frac{j}{N}}\;.$$

The upper limit is set by the Nyquist frequency, which means that the center frequency and its band edges should be below the Nyquist and the ½ power point of the anti-aliasing filter. A conservative estimate is

(75) $$f_{j\;max}=f_{tg}2^{\frac{j\;max}{N}}<\frac{f_{s}}{2}\Rightarrow j\;max<floor\left(Nlog_{2}\left[\frac{f_{s}}{2f_{tg}}\right]\right)\;.$$

The lower limit is set by the largest data window duration T

(76) $$f_{j\;min}=f_{tg}2^{\frac{j\;min}{N}}>\frac{2}{T}\Rightarrow j\;min>ceil\left(Nlog_{2}\left[\frac{2}{Tf_{tg}}\right]\right)$$

so that the center frequencies are defined by

(77) $$f_{j}=f_{c}2^{\frac{j}{N}},\;j\in \left[j\;min,\;j\;max\right]$$

which will be sufficient information to compute the Morlet scale ${𝓈}_{n}$. If one must convert to a sorted, monotonically increasing pseudoperiod, let

(78) $$\tau_{j}=\frac{1}{f_{j}},\;\tau_{0}=min\left(\tau_{j}\right)$$

and restart the counter for the period

(79) $$\tau_{n}=\tau_{0}2^{\frac{n}{N}},\;n\in \left[0,\;j\;max-j\min=length\;(f_{j}\right)]\;.$$

This re-indexing is much easier to do numerically than to describe algorithmically. For the purposes of illustration and demonstration, let us choose a signal frequency that exactly matches the target frequency; if this example fails there is no purpose in continuing. A sample rate of 200 Hz will be more than sufficient for this example. Gaussian noise with a standard deviation that is one bit below the signal standard deviation (factor of 1/2) is superposed, and then anti-alias filtered for all frequencies below Nyquist. The analytic function is computed numerically from the real pulse for later comparisons with the wavelet-reconstructed signal.

The CWT scalogram is computed using the complex nondimensional mother quantum wavelet of order *N*. The complex Gabor-Morlet wavelet in SciPy [25] is represented by the function scipy.signal.morlet2, and has the desired canonical form,

(80) ΨH(m)=1π14exp(−m22) exp(iMNm)

(81) $$\Psi_{u,n}\left(m\right)=\frac{1}{\sqrt{{𝓈}_{n}}}\Psi_{H}\left(\frac{m-u}{{𝓈}_{n}}\right)$$

(82) $${𝓈}_{n}={𝓈}_{0}\;2^{\frac{n}{N}}=\left[\frac{M_{N}}{2\pi }f_{s}\tau_{0}\right]\;2^{\frac{n}{N}}=\frac{M_{N}}{2\pi }\frac{f_{s}}{f_{n}}$$

(83) $$T_{n}=\left[M_{N}\tau_{0}\;\right]2^{\frac{n}{N}}=\frac{M_{N}}{f_{n}}$$

(84) $$M_{N}=2\sqrt{ln2}\;Q_{N}$$

(85) $$Q_{N}={\left[\;2^{\frac{1}{2N}}\;-\;2^{-\frac{1}{2N}}\right]}^{-1}\;.$$

The only free variables are the order *N*, the smallest time scale $\tau_{0}$, and the sample rate $f_{s}$. Although the nondimensionalized scale will change with the sample rate, the final results can always be returned to the physical domain frequencies $f_{n}$. The nominal number of points per window can be estimated from $f_{s}T_{n}$. The complex wavelet coefficients can be readily computed from the real part of the discrete version of the blast source-time function p(m)

(86) $$W_{n}\left[m\right]=\overset{Mp-1}{\underset{m^{\prime }=0}{\sum }}p\left(m^{\prime }\right)\Psi_{n}^{*}\left(m^{\prime }-m\right)=p⊛\Psi_{n}^{*}\left[m\right]$$

After minor conditioning, the SciPy CWT function [25] promptly invokes the convolution function. This is computationally expensive: we have turned a time series with Mp points into a complex 2[Mp x Nbands] array of band-passed waveforms. The terms wavelets and wavelet filter banks are often used interchangeably in the context of the CWT. 

**Figure 1:**
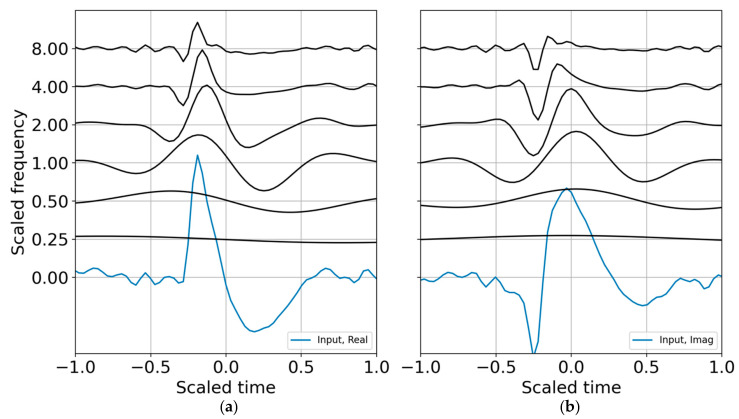
Analytic signal from mathematical equation, computation with SciPy Hilbert, and the continuous wavelet transfer (CWT) reconstruction. (**a**) Real part; (**b**) imaginary part. The wavelets were evaluated in binary bands (*N* = 1) and constructed around the target frequency of 6.3 Hz, which scales frequency and time. The real input waveform and its computed Hilbert transform are displayed in blue at the zero frequency.

**Figure 2:**
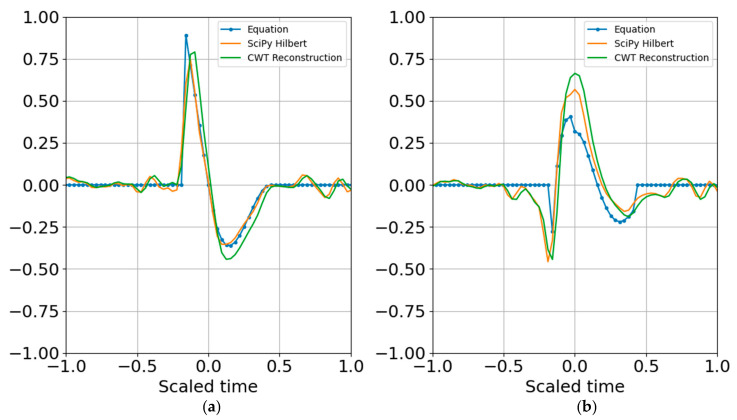
Wavelet reconstruction with binary bands. (**a**) Real part; (**b**) imaginary part. The Equation waveform has no noise and is not filtered, whereas Hilbert has Gaussian noise and has been anti-aliased filtered.

The wavelet-filtered reconstructed complex analytical signal can be approximated from

(87) $$g_{\mathbb{C}\;ij}\left[m_{k}:m_{l}\right]\approx \frac{\pi^{\frac{1}{4}}}{2}\;\overset{j}{\underset{n=i}{\sum }}\frac{W_{n}\left[m_{k}:m_{l}\right]}{\sqrt{{𝓈}_{n}}}$$

where the i, j indexes indicate that one may choose selected scales for the reconstruction over selected time indexes $m_{k}:m_{l}$ corresponding to the wavelet coefficients that best represent a signal of interest during the time interval of relevance. The wavelet CWT coefficients for the binary band decomposition are shown in Figure 1; the CWT coefficients are scaled by the reconstruction coefficients. A comparison of the input synthetic analytic record and the analytic signal reconstruction (summed over all scales) for the octave band representation is shown in Figure 2.

The reconstruction process recovers the original dimensionality of the time series but returns its Hilbert transform, so the total dimensionality may be doubled (2Mp sample points). If only the original real signal is desired, then the dimensionality is unchanged.

**Figure 3:**
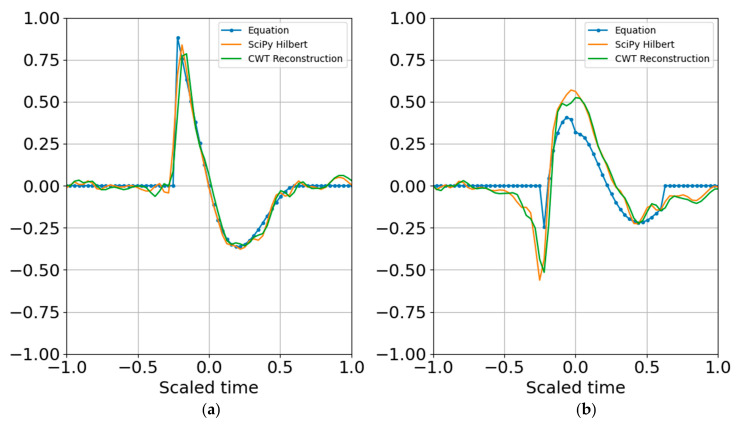
Wavelet reconstruction with 1/3 octave bands. (**a**) Real part; (**b**) imaginary part.

**Figure 4:**
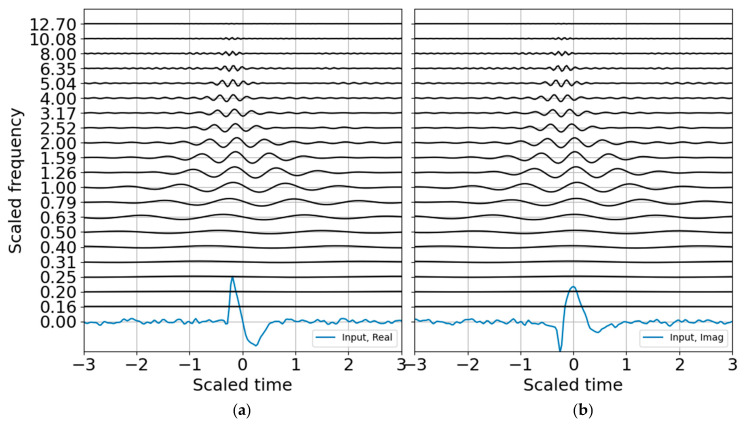
Wavelet decomposition with 1/3 octave bands, with CWT amplitudes scaled by the reconstruction coefficients. (**a**) Real part; (**b**) imaginary part. As with Figure 1, the input waveform is displayed at the zero frequency.

**Figure 5:**
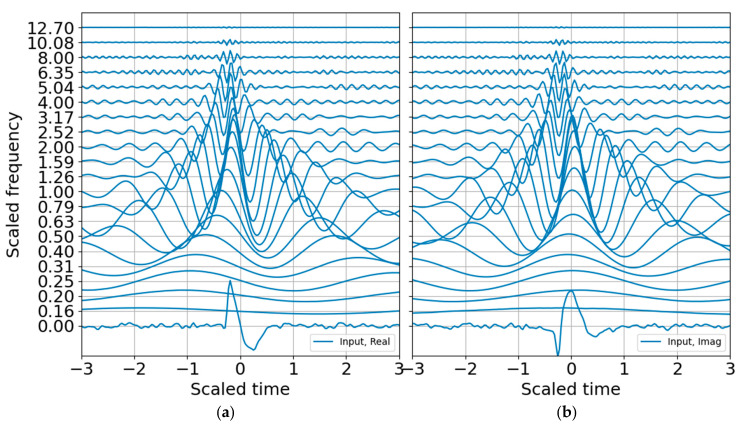
Wavelet decomposition in order 3 binary bands, raw CWT amplitudes. (**a**) Real part; (**b**) imaginary part.

The next steps estimate entropy and SNR, and consider sparse signal representation. Although binary bands are adequate for characterizing this signal, and are routinely used in the discrete wavelet transform, I take advantage of the flexibility offered by the CWT and use third order bands (*N* = 3) for the examples that follow. One of the benefits of third order bands is that the admissibility condition is better met and scales are recursive in powers of 2 and 10 ([1]). As presented in Appendix D, third order bands will contain over 99% of the Gabor box variance within an octave and within 80% of the full window $T_{n}$, reducing spectral leakage. If, in addition, one wants a factor of two accuracy in explosive yield estimates, 1/3 octave resolution is a minimum requirement. A third order band wavelet reconstruction is shown in Figure 3 and corresponds to the CWT decomposition presented in Figure 4. The wire mesh representation is the equivalent of the scalograms usually represented as color mesh plots, and illustrates the simplicity of the CWT decomposition. The primary difference between Figure 4 and Figure 5 is that the first scales the raw CWT coefficients by the reconstruction scaling, whereas Figure 5 shows the raw coefficients. 

**Figure 6:**
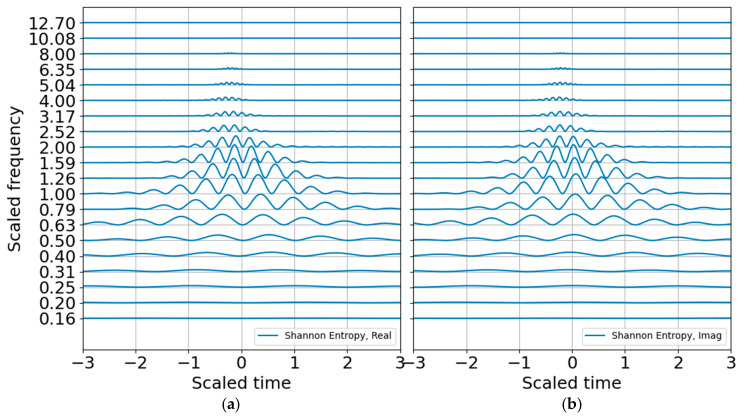
Shannon entropy in order 3 bands from raw CWT amplitudes. (**a**) Real part; (**b**) imaginary part.

The energy probability distribution is constructed from the wavelet coefficients to estimate entropy, as discussed in the previous section. The log energy entropy looks like any other scalogram and does not add much value, but the Shannon entropy plot is interesting and well scaled (Figure 6). The peak entropy is at the scaled blast center frequency of unity, as expected.

**Figure 7:**
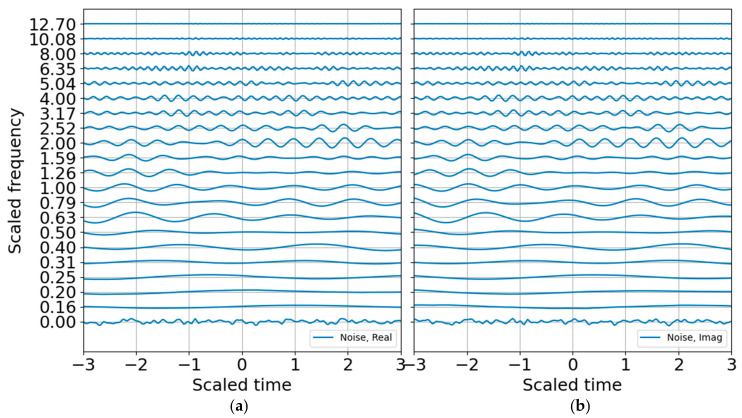
Raw CWT of noise in 1/3 octave bands. (**a**) Real part; (**b**) imaginary part.

Next a noise model is constructed to build the SNR and to establish criteria for standardized and reproducible sparse signal representation. Many are the ways to characterize noise, and few of them accurately characterize non-stationary noise over brief observation windows. An incorrect noise model can penalize the signal passband and degrade the signal SNR. For the white noise model with variance that is one bit below the signal variance, the CWT of the noise (Figure 7) shows how the high-frequency oscillations are adequately sampled whereas the low-frequency oscillations are undersampled. This leads to instability if the noise is only estimated over a brief observation record. In principle, one may build a noise model over a substantial time period to improve statistical significance under the assumption that the noise is statistically stationary. This can be a tenuous assumption in some circumstances. Noise studies are beyond the scope of this paper; the noise spectrum is flattened by using the mean of the noise coefficients to estimate the band-averaged noise level.

**Figure 8:**
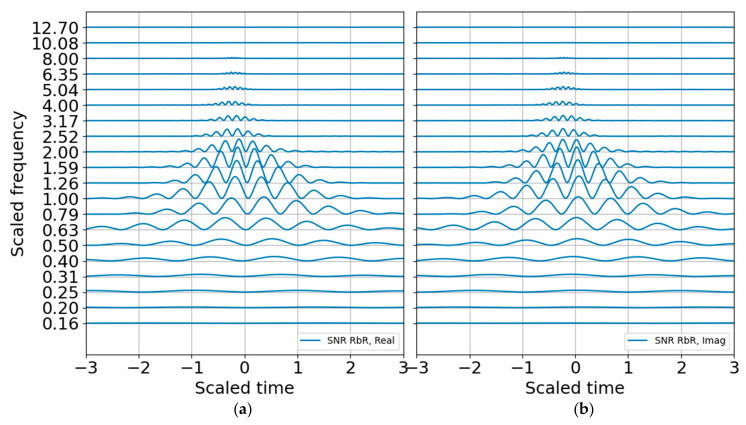
SNR RbR in 1/3 octave bands. (**a**) Real part; (**b**) imaginary part.

As anticipated, the binary SNR appears much like the log energy entropy since they are both scaled by a constant value, with the former over the band-averaged noise and the latter over the total energy. The SNR RbR, as described in the previous section, should also look very much like the entropy, except it would be zero for SNR of unity and positive for SNR > 1. The SNR RbR is shown in Figure 8 and indeed matches the Shannon entropy plot. This is encouraging; the entropy plot requires constructing an energy distribution that scales with the record, whereas the SNR requires constructing a noise model that is mostly independent of the record and should have more stability—as long as the ambient noise is approximately stationary or can at least be adequately modeled. If one is curating data for machine learning training, the entropy would be a good metric for picking and annotating possible signals as well as for refining noise models. If one is trying to trigger or detect signals operationally, the SNR may be a preferable metric since it makes no assumptions about the total energy in a record and only scales relative to a (preferably) stable noise representation.

**Figure 9:**
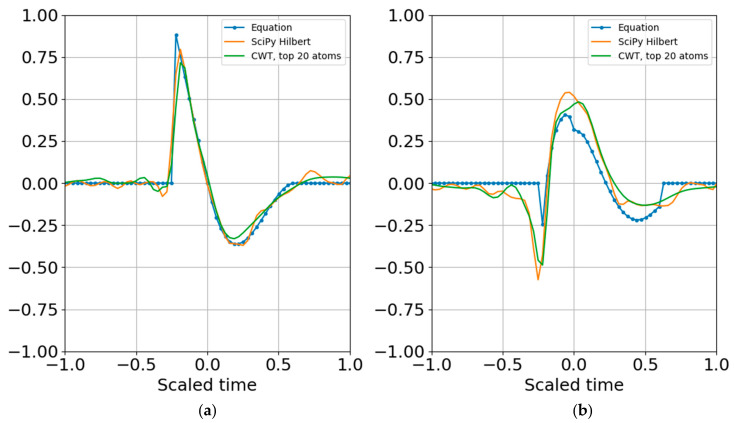
Superposition of largest SNR entropy coefficients per band using all twenty 1/3 octave bands. (**a**) Real part; (**b**) imaginary part. The noise standard deviation is one bit below the signal’s. Dimensionality is reduced to the number of coefficients and their corresponding time shifts.

**Figure 10:**
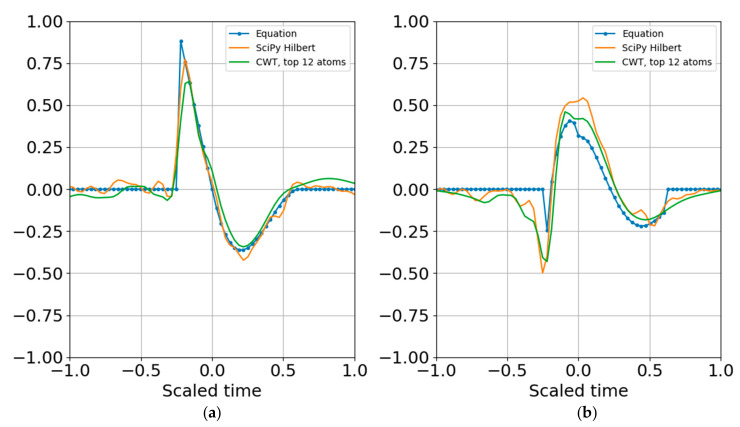
Superposition of largest coefficients per band within 4 bits of the peak SNR entropy. (**a**) Real part; (**b**) imaginary part. Dimensionality is further reduced by applying the cutoff.

One may use the CWT coefficient energy, the Shannon entropy, or the SNR RbR to test the feasibility of the sparse Gabor atom superposition. Suppose we use any of these Np scales x Mpoint time matrices to identify the peak contributions over the record, and define the complex time indexes as $m_{\mathbb{C}\;max}$. The quantum wavelet superposition would be expressed as

(88) $$g_{\mathbb{C}\;ij}\left[m_{k}:m_{l}\right]\approx \frac{\pi^{\frac{1}{4}}}{2}\;\overset{j}{\underset{n=i}{\sum }}\frac{W_{n}\left[m_{n\;\mathbb{C}\;max}\right]}{\sqrt{{𝓈}_{n}}}Re\left\{\Psi_{n}\left[m_{k}:m_{l}-m_{n\;\mathbb{C}\;max}\right]\right\}$$

where the dimensionality of the representation is reduced to the complex coefficients and time indexes. Since the wavelet function can be reproduced for any time index, the time array need not be stored. In other words, if there are 20 scales, there will be 20 real coefficients and time offsets and 20 imaginary coefficients and time offsets, with total dimensionality of 4 × 20 = 80 parameters. If there is sufficient SNR and the signal is band limited it is possible to further reduce dimensionality by removing any coefficients below a specified threshold that may be fitting to noise (e.g., overfitting). Figure 9 shows the result of reconstruction from the superposition of all the top atoms of the 20 scales, and Figure 10 shows reconstruction from a sparser set of 12 scales with the highest SNR RbR. Similar results were obtained using the Shannon entropy. The Gaussian noise standard deviation for these two runs was one bit below the signal standard deviation.

**Figure 11:**
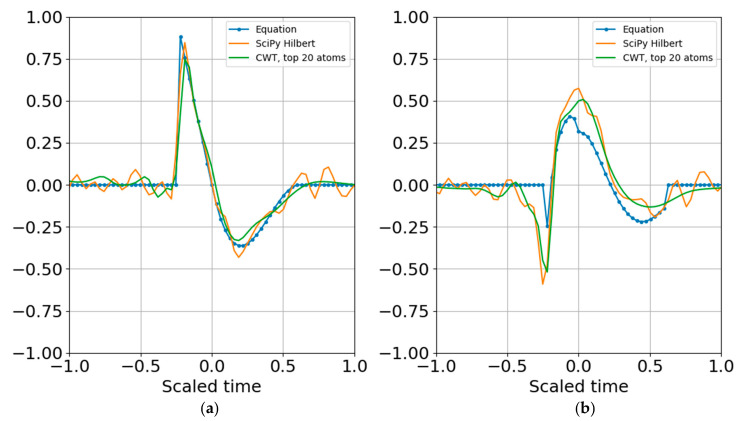
(**a**) Real part and (**b**) imaginary part of the original and reconstructed waveform. Increasing the noise amplitude so that its variance is the same as the signal variance still permitted reconstruction from the superposition of the largest atoms per band.

**Figure 12:**
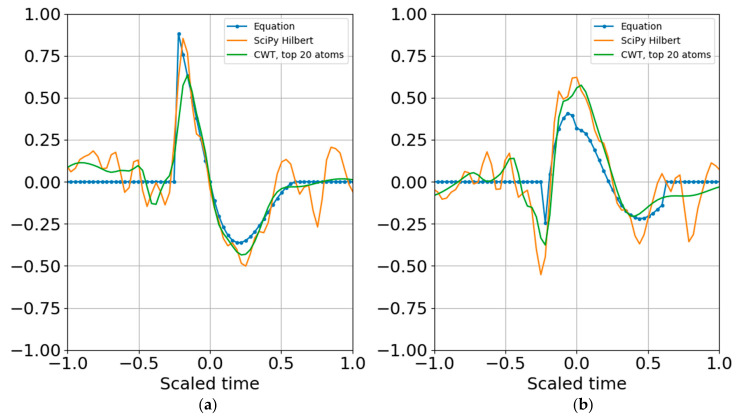
(**a**) Real part and (**b**) imaginary part of the original and reconstructed waveform. Increasing the noise standard deviation is one bit above the signal standard deviation also allowed reconstruction from the quantum wavelet superposition.

Increasing the noise standard deviation by a factor of two (one bit) still permits reconstruction from superposition (Figure 11), and increasing by another bit also allowed atomic reconstruction (Figure 12).

There is no end to the number of sensitivity studies that can be performed; in addition to other SNR tests, shifting the peak blast frequency away from the nominal target frequency still returned a stable reconstruction. Increasing the order past N > 6 only worsened the fit to the target waveform, increasing dimensionality and computational cost while decreasing reconstruction fidelity. This is expected from using a wavelet that does not match the target signature.

## 4. Concluding Remarks

This paper proposes a transition to binary metrics for digital data and introduces a standardized, quantized variation of the Gabor atoms with binary bases, optimal time-frequency resolution, and clear spectral energy containment. A binary entropy-like metric for the SNR is proposed and used to extract the peak coefficients to evaluate the performance of the superposition of Gabor atoms against the more traditional CWT reconstruction. Although the immediate application is the analysis of time series data collected with cyber-physical systems such as smartphones, the methods presented in this paper should be transportable to other types of digital records and can be extended to other wavelet families.

I used a synthetic pressure pulse corresponding to the detonation of one metric ton if TNT in Gaussian noise as an example, and did not include the blast amplitude as a key parameter in order to concentrate on the entropy and SNR, which are both dimensionless scaled quantities. Observations collected close to an explosion should have brief durations and a high SNR; for short pulses it is advisable to use Gabor atoms of small order (*N* = 1 − 6). Due to cube root yield scaling, the third order bands will provide a yield resolution—and uncertainty—of a factor of two, and one-sixth order bands will return square root of two yield resolution. In other words, the uncertainty of yield estimates obtained with the quantum wavelet would be inversely proportional to the cube root of the band order. Acceptable signal reconstructions were obtained from the CWT coefficients as well as the superposition of the peak third order Gabor atoms for the blast signature. At increasing distance from the source, the peak frequency is expected to drop [7] and the pulse disperses over time. This opens up the possibility for stable 6 and 12 order analyses with a corresponding improvement in yield resolution. Future work will concentrate on such dispersed signatures as well as consider other types of CW signatures that would be well matched to higher-order Gabor atoms.

The methods developed have the goal of providing a tunable, standardized framework for signature feature extraction that can be used for signal classification and which should be well suited for dictionary learning [13].

## Figures and Tables

**Table 1:** Quality factor *Q* and $M_{N}$ for standard fractional octave bands of order *N* ^1^.

*N*	$Q_{N}$	$M_{N}$
1	1.4142	2.3548
3	4.3185	7.1907
6	8.6514	14.4055
12	17.3099	28.8229
24	34.6235	57.6519
48	69.2488	115.3067
96	138.4984	230.6150

^1^ Dyadic base, G = 2.

**Table 2:** Exact and approximate quality factor *Q* for standard fractional octave bands of order *N* ^1^.

*N*	$Q_{N}$	$Q_{N}\;\approx \;\sqrt{2}N$
1	1.4142	1.4142
3	4.3185	4.2426
6	8.6514	8.4853
12	17.3099	16.9706
24	34.6235	33.9411
48	69.2488	67.8823
96	138.4984	135.7645

^1^ Dyadic base, G = 2.

**Table 3:** Approximate quality factor Q and $M_{N}$ for non-integer order *N* ^1^.

$Q_{N}$	$N\;\approx \;Q_{N}/\sqrt{2}$	$M_{N}$
1	0.7071	1.6651
2	1.4142	3.3302
4	2.8284	6.6604
8	5.6569	13.3209
16	11.3137	26.6417
32	22.6274	53.2835
64	45.2548	106.5670
128	90.5097	213.1340

^1^ Dyadic base, G = 2.

**Table 4:** Approximate quality factor *Q* and order *N* ^1^ for integer values of M

$M_{N}$	$\sim Q_{N}$	N
1	0.600561204	0.4246609
2	1.201122409	0.8493218
4	2.402244818	1.698643601
5	3.002806022	2.123304501
6	3.603367226	2.547965401
8	4.804489635	3.397287201

^1^ Dyadic base, G = 2.

## Disclaimer

This report was prepared as an account of work sponsored by agencies of the United States Government. Neither the United States Government nor any agency thereof, nor any of their employees, makes any warranty, express or implied, or assumes any legal liability or responsibility for the accuracy, completeness, or usefulness of any information, apparatus, product, or process disclosed, or represents that its use would not infringe privately owned rights. Reference herein to any specific commercial product, process, or service by trade name, trademark, manufacturer, or otherwise does not necessarily constitute or imply its endorsement, recommendation, or favoring by the United States Government or any agency thereof. The views and opinions of authors expressed herein do not necessarily state or reflect those of the United States Government or any agency thereof. The United States Government is authorized to reproduce and distribute reprints for Governmental purposes notwithstanding any copyright notation thereon.

## Appendix A. Generalized Constant Q Bands

This work builds on the Infrasonic Energy, Nth Octave (Inferno) framework [1], which has been implemented in infrasound array processing algorithms for nuclear monitoring applications [3,4] Logarithmic constant-bandwidth, also referred to as proportional frequency or constant quality factor (Q) bands, are traditionally defined by their scaled bandwidth

(A1) $$\frac{\Delta f}{f_{n}}=\frac{f_{H}-f_{L}}{f_{n}}=\;\frac{1}{Q}$$

where $f_{n}$ is the center frequency of band number *n* and $f_{H}$ and $f_{L}$ are referred to as the upper and lower band edge frequency, respectively. Defining the center, upper, and lower band edge *periods* $\tau_{n},\;\tau_{H},\;and\;\tau_{L}\;$ as

(A2) $$\tau_{n}=\frac{1}{f_{n}},\;\tau_{H}=\frac{1}{f_{L}},\;\tau_{L}=\frac{1}{f_{H}},$$

the

(A3) $$\frac{\Delta \tau }{\tau_{n}}=\frac{\tau_{H}-\tau_{L}}{\tau_{n}}=\frac{\Delta f}{f_{n}}=\frac{\Delta \omega }{\omega_{n}}=\;\frac{1}{Q}\;.\;$$

In this section we generalize the constant-Q framework to the logarithmic discretization of evaluation intervals relative to a given reference scale and base. For a given reference scale $\tau_{0}$, which could be time, frequency, spatial length, wavenumber, or any other useful metric, we define a logarithmic scale base *G* > 1 and center scale $\tau_{n}$ as

(A4) $$\frac{\tau_{n}}{\tau_{0}}=G^{\frac{n}{N}}$$

where *n* is the band number and *N* is the band order, subject to the constraints n∈ℤ,  N≥1.

The natural base for both contemporary and quantum computers is base 2, and analysis windows with powers of two are recommended for complex computations at large scales. Many efficient algorithms are based on binary (base two) filter banks. Selecting *G* = 2 yields

(A5) $$\frac{\tau_{n}}{\tau_{0}}=2^{\frac{n}{N}},\frac{\tau_{H}}{\tau_{n}}=2^{\frac{1}{2N}},\frac{\tau_{L}}{\tau_{n}}=2^{-\frac{1}{2N}},\frac{\tau_{H}\tau_{L}}{\tau_{n}^{2}}=1$$

(A6) $$Q_{N}={\left[\;2^{\frac{1}{2N}}\;-\;2^{-\frac{1}{2N}}\right]}^{-1}.$$

Note that center and band edge scales attached to a given band *n* change with the order *N*, reference scale $\tau_{0}$, and the reference base *G*. If the reference scale and base are standardized, all bands are invariant. For example, the concert A pitch standard is fixed at 440 Hz and may be used to tune other instruments anywhere and at any time.

The next step substantially simplifies the estimation of constant-Q bands with a minimal introduction of a 2% computational error. To the author’s knowledge, this is the first time this expression is presented (and he would be most grateful to be informed otherwise). Numerical evaluation shows that

(A7) $$\underset{N\to \infty }{\lim}\frac{Q_{N}}{Q_{1}}=\underset{N\to \infty }{\lim}\left(\;G^{\frac{1}{2}}\;-\;G^{-\frac{1}{2}}\right){\left(\;G^{\frac{1}{2N}}\;-\;G^{-\frac{1}{2N}}\right)}^{-1}\approx N\frac{G-1}{\sqrt{G}ln\left(G\right)}\approx \left(1.02\;\right)N\approx N$$

(A8) $$Q_{N}\approx NQ_{1}=N\left[\frac{\sqrt{G}}{G-1}\right]$$

The center frequencies and band edges, and thus the quality factor, of traditional fractional octave bands are well known and can be readily computed for all the standard bands. The primary value of the expression for $Q_{N}$ is that it provides a simple, explicit estimate of the relation between the quality factor and the band order, which in turn permits an estimate of the support window duration for a given wavelet in terms of the band order. Numerical inspection shows that for most practical applications and for $G=2\approx 10^{\frac{3}{10}}$, even those when N is non integer, we can use the expression

(A9) $$Q_{N}=\frac{f_{n}}{\Delta f_{n}}\approx \sqrt{2}N$$

to estimate the relationship between the band order and the quality factor.

Although the center frequency is traditionally defined as the geometric mean of the band edges, the ½ power spectral points at the band edges are only symmetric around the arithmetic mean of the center frequency. The relation between the arithmetic mean $f_{na}=\left(f_{L}+f_{H}\right)/2$ and the geometric mean $f_{ng\;}=\sqrt{f_{L}f_{H}}$ of the center frequency of fractional binary bands is

(A10) $$\frac{f_{na}}{f_{ng\;}}\;≅\sqrt{1+\;\frac{1}{8N^{2}}}\approx 1+\;\frac{1}{16N^{2}}$$

where the approximation uses the binomial expansion. The arithmetic and geometric center frequencies are close to each other, and for fractional octave bands (N>1) get ever tighter. However, the band edge power levels at the half band width $\Delta f_{n}/2$ should be considered to be relative to the arithmetic mean rather than the geometric mean. In general practice it is easier to use the arithmetic frequency as $f_{n}$, with the understanding that the fractional octave specifications are defined by geometric scaling.

As an extension of the Inferno framework [1] the nominal duration of the Gabor atom window $T_{n}$ may be defined as a multiple $M_{N}$ of the scale as

(A11) $$T_{n}\left(N,n\right)≝M_{N}\tau_{n}=M_{N}\tau_{0}G^{\frac{n}{N}}$$

where the scale multiplier $M_{N}$ is set by the half power points of the wavelet. Traditional constant-Q frameworks in acoustics and music applications match the 12-tone equal temperament system (N = 12) for G=2 or $G=10^{\frac{3}{10}}\approx 2$ and are consistent with the Renard series recommended in ISO3 for N=1, 3, 6, 12, 24.

## Appendix B. The Gabor Atom

Different disciplines call the same things different names; many of the challenges in present-day data science are often due to divergent lexicon and the diversity of applications specific to each field. The idea of using a windowed sinusoid as a basis function for signal representation was developed in detail in Gabor’s [2] landmark paper, where he also introduced the time-frequency uncertainty principle. Gabor’s atoms were further developed by Grossman and Morlet [14] and P. Goupillaud et al. [15] (amongst others), who formalized and popularized what we now know as *wavelet* transforms. Mallat [13] presents a lucid overview of the complementary nature of Fourier and wavelet representations in his Wavelet Tour of Signal Processing; the serious student would be wise to consider it required reading.

The Gabor wavelet is a special case of a wavelet-modulated window ([13] Equations (4.60)–(4.62)) and is representative of a bandwidth-limited compressed pulse [12]. For a physical scientist, its most intuitive form is

(A12) Ψ(x)=1[πσ2]14exp(−x22σ2) exp(iηn x),

representing a sinusoid with scaled time $x=\;f_{s}t$ and scaled angular frequency $\eta_{n}\;$(or linear space and wavenumber) modulated by Gaussian window with standard deviation σ. Comparison with the canonical expression

(A13) Ψn(x)=1(π𝓈n2)14exp{−12[x𝓈n]2}exp{i[2πfn fs]x}

shows that the scaled angular frequency and standard deviation are

(A14) $$\eta_{n}=\frac{2\pi f_{n}}{\;f_{s}},\sigma ={𝓈}_{n},\sigma \eta =M_{N}\frac{f}{f_{n}}$$

The Fourier transform of the mother wavelet is

(A15) Ψ^(η)=[4πσ2]14 exp{−12σ2[η−ηn]2}=[4π𝓈n2]14 exp{−12MN2[ffn−1]2},

has unit second moment

(A16) $$\int_{-\infty }^{\infty }\Psi \left(x\right)\Psi^{*}\left(x\right)dx=1,$$

and it first moment vanishes in the limit

(A17) $$\int_{-\infty }^{\infty }\Psi \left(t\right)dt\;\to 0\;\mathrm{for}\;\sigma^{2}\eta_{c}{}^{2}\gg 1.$$

Another important representation of the Gabor wavelet [26,27] is

(A18) $$\psi ={\left(4\pi \sigma^{2}\right)}^{-\frac{1}{4}}\;\Psi$$

(A19) $$\psi \left(x\right)=\frac{1}{{\left[2\pi \sigma^{2}\right]}^{\frac{1}{2}}}\;exp\left\{-\frac{x^{2}}{2\sigma^{2}}\right\}exp\left\{i\eta_{c}x\right\}$$

with the advantage that its Fourier transform

(A20) $$\hat{\psi }\left(\eta \right)=\;exp\left\{-\frac{1}{2}\sigma^{2}{\left[\eta -\eta_{c}\right]}^{2}\right\}=\;exp\left\{-\frac{1}{2}M_{N}^{2}{\left[\frac{f}{f_{n}}-1\right]}^{2}\right\}.$$

has a peak amplitude of unity and yields equal-amplitude filter banks.

The Inferno framework was developed with the introduction of multiresolution array processing in the field of infrasound. The time duration of an analysis window at a specific period is represented as

(A21) $$T_{n}=M_{N}\;\tau_{n}\;.$$

This time window generally sets the temporal resolution of the resulting data products. In the case of the STFT, the fixed-duration analysis window can be referred to as the window of integration. In other words, the integration window $T_{n}$ is defined as a multiple $M_{N}$ of the pseudo period. This window immediately constrains the lowest frequency $f_{min}$ that can be represented and the resolution of a spectral representation,

(A22) $$f_{min}=\frac{1}{T_{n}}\;.$$

The upper bandwidth of the analysis window can be set by the Nyquist frequency, which is half of the sampling frequency of the digital time series. In practice the upper bandwidth is close to one quarter of Nyquist. Although this representation is simple and tidy, it is not particularly informative. A more useful representation of window duration is the number of wavelet oscillations in the window, which can be estimated from the quality factor $Q_{N}$ of the wave function. As presented in Appendix C, the relation between the scale multiplier $M_{N}$ and the quality factor can be estimated by the ½ power (−3 dB, or half bit) points on the power spectrum,

(A23) $$M_{N}=2\sqrt{ln2}\;Q_{N}\;.$$

The wavelet admissibility condition for the for this wavelet is equivalent to the zero mean, or

(A24) $$M_{N}^{2}\;\gg 1$$

which is essentially met by the standard bands presented in Table 1. Although traditionally the Nth octave frequencies are represented by the geometric mean of the band edge frequencies (Appendix A), in the evaluation of spectral power losses it is important to use the arithmetic mean for $f_{n}$ which would be centered in the bandwidth $\Delta f_{n}$ in linear frequency space. Since the ratios of the arithmetic and geometric means are constant and set by the band order *N*, the geometric scaling is still preserved.

The canonical form for computational evaluation is:

(A25) Ψn(x−x′)=1π141𝓈nexp{−12[x−x′𝓈n]2}exp{iMN[x−x′𝓈n]} .

The second b-type form has a different structure

(A26) $$\psi_{n}\left(x\right)\;={\;\Psi }_{x^{\prime },n}\left(x\right){\left(4\pi \right)}^{-\frac{1}{4}}\;{𝓈}_{n}^{-\frac{1}{2}}$$

(A27) $$\psi_{n}\left(x-x^{\prime }\right)={\left(2\pi \right)}^{-\frac{1}{2}}\;{𝓈}_{n}^{-1}\;exp\left\{-\frac{1}{2}{\left[\frac{x-x^{\prime }}{{𝓈}_{n}}\right]}^{2}\right\}exp\left\{i2\pi \frac{\;f_{n}}{\;f_{s}}\left(x-x^{\prime }\right)\right\}$$

applying

(A28) $${𝓈}_{n}=\;{𝓈}_{0}\;2^{\frac{n}{N}},$$

yields

(A29) $$\psi_{n}\left(x-x^{\prime }\right)={\left(\pi \;2{𝓈}_{o}^{2}\right)}^{-\frac{1}{2}}\;{\left[s_{n}\right]}^{-1}\;exp\left\{-\frac{1}{2{𝓈}_{o}^{2}}{\left[\frac{x-x^{\prime }}{s_{n}}\right]}^{2}\right\}exp\left\{i\frac{M_{N}}{{𝓈}_{0}}\left[\frac{x-x^{\prime }}{s_{n}}\right]\right\}$$

which has the form

(A30) $$\psi_{N}\left(\mu \right)=\frac{1}{\sqrt{\pi b}}\;exp\left\{-\frac{\mu^{2}}{b}\right\}exp\left\{i\frac{M_{N}}{{𝓈}_{0}}\;\mu \right\}$$

(A31) $$\psi_{n}\left(\mu \right)=\frac{1}{s_{n}}\psi_{N}\left(\frac{\mu -\mu^{\prime }}{s_{n}}\right)$$

with

(A32) $$s_{n}=\frac{{𝓈}_{n}}{{𝓈}_{0}}\;=2^{\frac{n}{N}},n\geq 0,\;{𝓈}_{0}=M_{N}\frac{\;f_{s}\tau_{0}}{2\pi }$$

(A33) $$b=2{𝓈}_{o}^{2}=2{\left[M_{N}\frac{\;f_{s}\tau_{0}}{2\pi }\right]}^{2}.$$

Note that since

(A34) $$b=8ln2{\left(\frac{\;f_{s}}{\Delta \omega_{0}}\right)}^{2}$$

the “bandwidth” b is inversely proportional to the actual bandwidth of the highest frequency.

## Appendix C. The Q of the Quantum Wavelet

The power spectral density of the Gabor wavelet is:

(A35) Ψ^2n(f)=[4π𝓈n2]12 exp{−MN2[f−fnfn]2},

(A36) $$\frac{{\hat{\Psi }}^{2}{}_{u,n}\left(f_{n}\;\pm \;\Delta f_{n}/2\right)}{{\hat{\Psi }}^{2}{}_{u,n}\left(f_{n}\right)}=\;exp\left\{-M_{N}^{2}{\left[\frac{\Delta f_{n}}{2f_{n}}\right]}^{2}\right\}=exp\left\{-{\left[\frac{M_{N}}{2Q_{N}}\right]}^{2}\right\}=\frac{1}{Y}$$

where Y is the fractional power loss. There exist various definitions of the quality factor of a system. This paper defines $Q_{N}$ by 1/2 of the spectral power relative to the peak spectral power, where Y=2. Therefore, for the Gabor wavelet,

(A37) $$M_{N}=2\sqrt{ln2}\;Q_{N}\;.$$

Consider the decay of the spectrum relative with distance δ from the peak frequency

(A38) $$\frac{{\hat{\Psi }}^{2}{}_{u,n}\left(f_{n}+\;\delta \Delta f_{n}/2\right)}{{\hat{\Psi }}^{2}{}_{u,n}\left(f_{n}\right)}\;=exp\left\{-{\left[\frac{\delta M_{N}}{2Q_{N}}\right]}^{2}\right\}=exp\left\{-{\left[\delta \sqrt{ln2}\right]}^{2}\right\}=2^{-\delta^{2}}.$$

The loss in dBs and binary bits can be expressed as

(A39) $$dB=\;10*log_{10}(2^{-\delta^{2}})=\;-\delta^{2}10*\log_{10}\left(2\right)\approx -3\delta^{2}$$

(A40) $$bR=\;\frac{1}{2}log_{2}(2^{-\delta^{2}})=\;\frac{-\delta^{2}}{2}\;.$$

There is a loss of 3 dB, 12 dB, 27 dB, and 48 dB, and a binary power loss of ½, 2, 4.5, and 8 fbits, for integer multiples of the bandedge δ=1, 2, 3, 4, respectively.

It is worth considering an alternate definition for the quality factor of an oscillator. Consider the time required for the amplitude to drop to 1/e of its peak value. In the case of the Quantum wavelet this is set by the Gaussian envelope, and this particular definition is best suited for the real part of the wavelet which is symmetric about the origin. By applying this definition,

(A41) $$exp\left\{-\frac{1}{2}{\left[\frac{x}{{𝓈}_{n}}\right]}^{2}\right\}=exp\left\{-\frac{1}{2}{\left[\frac{f_{s}\tau_{e}}{{𝓈}_{n}}\right]}^{2}\right\}=exp\left\{-\frac{1}{2}{\left[\frac{\omega_{n}\tau_{e}}{M_{N}}\right]}^{2}\right\}=exp\left\{-1\right\}$$

(A42) $$\tau_{e}=\frac{\sqrt{2}}{\pi }\frac{T_{n}}{2}\approx 0.45\frac{T_{n}}{2}\;.$$

Since the wavelet is symmetric, this states that the portion of the wavelet contained within 2$\tau_{e}$ of the window has an amplitude above 1/e of the peak. The quality factor associated with this type of oscillator is

(A43) $$\;Q_{e}=\frac{\omega_{n}\tau_{e}}{2}=\frac{M_{N}}{\sqrt{2}}$$

and comparison with the half power point quality factor shows

(A44) $$Q_{e}=\sqrt{2ln2}\;Q_{N}\approx 1.1774\;Q_{N}$$

and they are sufficiently close to each other to be equivalent for descriptive purposes. The time duration of the quantum wavelet is defined by

(A45) $$T_{n}=M_{N}\;\tau_{n}=2\sqrt{ln2}Q_{N}\tau_{n}$$

where $\;Q_{N}\approx \;Q_{e}$ can be interpreted as the number of oscillations in a little less than half of the total window $T_{n}$ with amplitude above 1/e of the maximum amplitude. The remaining half of the window is useful to allow the wavelet to settle down and meet the desirable condition of a vanishing first moment.

Practical implementations of Gabor wavelets and their variants often have to make some compromises in the application of the wavelet duration $T_{n}$, especially if the window is required to be a power of two. Direct integration of the wavelet power over the window $T_{n}$ shows that it contains 99.999% of all the power. Integration over $2\tau_{e}$ will be insufficient (Appendix D). However, there exists a third quality factor defined by

(A46) $$exp\left\{-\frac{1}{2}{\left[\frac{\omega_{n}\tau_{\pi }}{M_{N}}\right]}^{2}\right\}=exp\left\{-\pi \right\}$$

where

(A47) $$\;Q_{\pi }=\frac{\omega_{n}\tau_{\pi }}{2}$$

(A48) $$\;Q_{\pi }=\sqrt{\pi }Q_{e}\approx 1.7724\;Q_{e}$$

(A49) $$\;\tau_{\pi }=\sqrt{\pi }\tau_{e}=\sqrt{\frac{2}{\pi }}\frac{T_{n}}{2}\approx 0.7978\frac{T_{n}}{2}\;.$$

In other words, $2\tau_{\pi }$ encompasses ~80% of the window, and integration of the wavelet power over $2\tau_{\pi }$ returns 99.96% of the total power. Therefore $2\tau_{\pi }=0.8T_{n}$ may be a reasonable lower bound for the wavelet duration. This is further considered in Appendix D.

## Appendix D. The Gabor Box

Gabor introduced the time-frequency uncertainty principle in his landmark paper [2]. It is not possible to observe for all time and reach zero frequency. It is also impossible to sample infinitely fast and reach infinite frequency. All observations require a restriction in the observation time and the observation rate, and this places hard limits on the observable bandwidth of a process. The fundamental discretization interval scale invokes the Gabor uncertainty principle, which states the time and period of a signal cannot be known exactly but can be contained inside the box defined by the temporal and frequency variance of the probability distribution of the wave function.

This section follows the generalized mathematical formalism of ([13], Section 2.3.2, Uncertainty Principle). As in [13] and [7] the Fourier Transform pair used in this work is

(A50) $$\hat{f}\left(\omega \right)=\int_{-\infty }^{\infty }f\left(t\right)e^{-j\omega t}dt$$

(A51) $$f\left(t\right)=\frac{1}{2\pi }\int_{-\infty }^{\infty }\hat{f}\left(\omega \right)e^{j\omega t}d\omega ,$$

where $\hat{f}\left(\omega \right)$ and f(t) may be complex, and t and ω are nondimensionalized time Equation (13). The Parseval-Plancherel identity asserts that

(A52) $$\|f\|{}^{2}=\int_{-\infty }^{\infty }{\left|f\left(t\right)\right|}^{2}dt=\frac{1}{2\pi }\int_{-\infty }^{\infty }{\left|\hat{f}\left(\omega \right)\right|}^{2}d\omega =\;\frac{1}{2\pi }\|\hat{f}\|{}^{2}$$

Where

(A53) $${\left|f\right|}^{2}=f\cdot f^{*}$$

and the asterisk denotes complex conjugation. A related identity is routinely used in Fourier and Wavelet analyses and the application of filter banks

(A54) $$\int_{-\infty }^{\infty }f\left(t\right)g^{*}\left(t\right)dt=\frac{1}{2\pi }\int_{-\infty }^{\infty }\hat{f}\left(\omega \right){\hat{g}}^{*}\left(\omega \right)d\omega \;.$$

The Gabor uncertainty principle constrains uncertainty to Gabor box defined by the variance in time and frequency. It is equivalent to the Heisenberg uncertainty principle for position and momentum extended to time and frequency, or space and wavenumber. Let a one-dimensional signal of interest be represented by a wave function f(t). The probability density that a signal can be localized in time at a given time t is

(A55) $$\frac{{\left|f\left(t\right)\right|}^{2}}{\|f\|{}^{2}}=\frac{2\pi {\left|f\left(t\right)\right|}^{2}}{\|\hat{f}\|{}^{2}},$$

and the probability density that its angular frequency is ω is

(A56) $$\frac{{\left|\hat{f}\left(\omega \right)\right|}^{2}}{\|\hat{f}\|{}^{2}}=\frac{{\left|\hat{f}\left(\omega \right)\right|}^{2}}{2\pi \|f\|{}^{2}}.$$

The variance in the time localization of the signal as

(A57) $$\sigma_{t}^{2}=\frac{1}{\|f\|{}^{2}}\int_{-\infty }^{\infty }{\left(t-u\right)}^{2}\;{\left|f\left(t\right)\right|}^{2}dt.$$

and the variance in the frequency localization of the signal as

(A58) $$\sigma_{\omega }^{2}=\frac{1}{\|\hat{f}\|{}^{2}}\int_{-\infty }^{\infty }{\left(\omega -\xi \right)}^{2}\;{\left|\hat{f}\left(\omega \right)\right|}^{2}d\omega .$$

Reference [13] uses these expressions to rederive the Heisenberg-Gabor uncertainty principle, which states that the temporal and angular frequency variance satisfy:

(A59) $$\sigma_{t}^{2}\sigma_{\omega }^{2}\geq \frac{1}{4}\;.$$

In the special case of the Gabor-Morlet wavelet and its Quantum spawn, where the wave function is symmetric and centered around the time-shift u and the spectrum is symmetric relative to the peak frequency $\omega_{n},$ the variance for the time and frequency distribution of the signal wave function can be readily evaluated.

(A60) $$\sigma_{t}^{2}=\frac{1}{\|\psi_{H}\|{}^{2}}\int_{-\infty }^{\infty }{\left(t-u\right)}^{2}\;{\left|\psi_{H}\left(t-u\right)\right|}^{2}dt=\;\frac{1}{2}{𝓈}_{n}^{2}$$

(A61) $$\sigma_{\omega_{n}}^{2}=\frac{1}{\|{\hat{\psi }}_{H}\|{}^{2}}\int_{-\infty }^{\infty }{\left(\omega -\omega_{n}\right)}^{2}\;{\left|{\hat{\psi }}_{H}\left(\omega -\omega_{n}\right)\right|}^{2}d\omega =\;\frac{1}{2}{𝓈}_{n}^{-2}$$

and the Gabor box defined by the variance is minimal,

(A62) $$\sigma_{t}^{2}\sigma_{\omega_{n}}^{2}=\frac{1}{4},$$

which is another reason for this wavelet’s popularity. Equations (A60) and (A61) are converted to physical time in Equation (22) of the main text.

Consider the standard deviation for time integrated over the scaled window $\epsilon T_{n}$

(A63) $$\sigma_{t}^{2}\left(\epsilon \right)=\frac{1}{\|\psi_{H}\|{}^{2}}\int_{u-\frac{\epsilon T_{n}}{2}}^{u+\frac{\epsilon T_{n}}{2}}{\left(t-u\right)}^{2}\;{\left|\psi_{H}\left(t-u\right)\right|}^{2}dt=\;\frac{1}{\sqrt{\pi }}{\left[\frac{M_{N}}{\omega_{n}}\right]}^{2}\int_{-\epsilon \pi }^{\epsilon \pi }x^{2}\;e^{-x^{2}}\;dx$$

(A64) $$\int_{-a}^{a}x^{2}\;e^{-x^{2}}\;dx=\frac{\sqrt{\pi }}{2}\left[erf\left(a\right)-\frac{2}{\sqrt{\pi }}a\;e^{-a^{2}}\right]\;.$$

For $\epsilon \geq \frac{3}{2\pi }$

(A65) $$\sigma_{t}^{2}\left(\epsilon \right)≅\;\frac{1}{2}{𝓈}_{n}^{2}\;erf\left(\epsilon \pi \right)\;.$$

For ϵ=[1.0, 0.8, 0.45] 

(A66) $$\sigma_{t}^{2}\left(\epsilon \right)≅\;\frac{1}{2}{𝓈}_{n}^{2}\left[0.9999,\;0.9996,\;0.9544\;\right]\;,$$

where ϵ corresponds to integration over $T_{n},\;2\tau_{\pi }\approx 0.8T_{n},\;and\;2\tau_{e}\approx 0.45T_{n}$, corresponding to the full window, the decay time associated with $Q_{\pi }$, and the e-folding time associated with $Q_{e}$, respectively (Appendix C).

Next, consider the standard deviation for time integrated over the scaled window $\epsilon T_{n}$

(A67) $$\sigma_{\omega_{n}}^{2}\left(\delta \right)=\frac{1}{\|{\hat{\psi }}_{H}\|{}^{2}}\int_{\omega_{n}-\frac{\delta \Delta \omega_{n}}{2}}^{\omega_{n}+\frac{\delta \Delta \omega_{n}}{2}}{\left(\omega -\omega_{n}\right)}^{2}\;{\left|{\hat{\psi }}_{H}\left(\omega -\omega_{n}\right)\right|}^{2}d\omega =\;=\;\frac{1}{\sqrt{\pi }}{\left[\frac{M_{N}}{\omega_{n}}\right]}^{-2}\int_{-\delta \sqrt{ln2}}^{\delta \sqrt{ln2}}x^{2}\;e^{-x^{2}}\;dx$$

(A68) $$\sigma_{\omega_{n}}^{2}\left(\delta \right)≅\;\frac{1}{2}{𝓈}_{n}^{-2}\;\left[erf\left(\delta \sqrt{ln2}\right)-\frac{2}{\sqrt{\pi }}\left(\delta \sqrt{ln2}\right)\;2^{-\delta^{2}}\right]\;.$$

For δ=[1,2,3,4] 

(A69) $$\sigma_{\omega_{n}}^{2}\left(\delta \right)≅\;\frac{1}{2}{𝓈}_{n}^{-2}\left[0.2912,\;0.8640,\;0.9941,\;0.9999\right]$$

where δ corresponds to integration over $\Delta \omega_{n},\;2\Delta \omega_{n},\;3\Delta \omega_{n},\;and\;4\Delta \omega_{n}$, respectively. These results show that the Gabor box can be well approximated (>99% of the variance) by a window of duration $2\tau_{\pi }=0.8T_{n}$ and a bandwidth of $3\Delta \omega_{n},$ and over 99.99% of the variance is contained by a Gabor box of dimensions $T_{n},\;4\Delta \omega_{n}$. In other words, third octave bands will contain over 99% of the variance within its octave and within 80% of the full window $T_{n}$.

## Appendix E. The Gabor Family

A few variations of the Gabor-Morlet wavelet are available in present-day computing environments. One of the more familiar forms of the mother wavelet used in modern computations [26,27] is

(A70) $$\psi \left(\mu \right)=\frac{1}{\sqrt{\pi b}}\;exp\left\{-\frac{\mu^{2}}{b}\right\}exp\left\{i2\pi {\bar{f}}_{b}\;\mu \right\}$$

(A71) $$\psi_{\mu^{\prime },n}\left(t\right)=\frac{1}{s_{n}}\psi \left(\frac{\mu -\mu^{\prime }}{s_{n}}\right)\;.$$

This form is found in the Matlab “cmor” function as well as the Python Pywavelets [28] “cmorB-C” function with $C={\bar{f}}_{b}$. The term b is referred to as the “bandwidth parameter” of the wavelet. The Quantum wavelet has the equivalence

(A72) $$s_{n}=\;2^{\frac{n}{N}},n\geq 0,$$

(A73) $$\tau_{n}=\tau_{0}s_{n}=\frac{1}{f_{0}}s_{n}$$

(A74) $$b=2{\left[M_{N}\frac{\;f_{s}\tau_{0}}{2\pi }\right]}^{2}$$

(A75) $$C={\bar{f}}_{b}=\frac{\;f_{0}}{\;f_{s}}=\frac{1}{\;f_{s}\tau_{0}}$$

where $f_{0}$, the highest center frequency, is used as the starting point. The scaled wavelet duration is $M_{N}\frac{f_{s}}{f_{n\;}}$ and can be rounded to approximate the number of points for each scale.

Foster [29] expresses the abbreviated Morlet wavelet as

(A76) $$F\left(z\right)=e^{iz-cz^{2}}=exp\left\{i\omega_{n}t-\frac{1}{2M_{N}^{2}}\omega_{n}^{2}t^{2}\right\}$$

so that $z=\omega_{n}t$ and now $c=\frac{1}{2M_{N}^{2}}$ is inversely proportional to the Q of the wave function. The beauty of Foster’s approach is that it can be used for unevenly sampled data. A modernization of this algorithms can be found at [30].

## Appendix F. The Analytic Function of a Blast Pulse

The reconstruction coefficients of the complex Morlet CWT return the imaginary part of the analytic signal. The complex analytic signal corresponding to the real signal $g\left(\hat{\tau }\right)$ is

(A77) $$g_{\mathbb{C}}\left(\hat{\tau }\right)=g\left(\hat{\tau }\right)+j\;ℋ\left[g\left(\hat{\tau }\right)\right]$$

where ℋ denotes the Hilbert transform, a recurrent topic in wave propagation as reflection introduces phase shifts that are often modeled as Hilbert transforms of the original signal [31]. For example, some of the U-shaped infrasound waveforms associated with thermospheric returns resemble the Hilbert transform of an explosion pulse [3]. The Hilbert transform is also useful for estimating instantaneous frequency and in the computation of the Hilbert-Huang transform [32].

Let $g\left(\hat{\tau }\right)$ represent the Granström Triangular (GT) pulse [7],

(A78) $$g\left(\hat{\tau }\right)=\left(1-\hat{\tau }\right),\;0\leq \hat{\tau }\leq 1$$

(A79) $$g\left(\hat{\tau }\right)=\frac{1}{6}\left(1-\hat{\tau }\right){\left(1+\sqrt{6}-\hat{\tau }\right)}^{2},\;1<\hat{\tau }\leq 1+\sqrt{6}\;.$$

The Hilbert transform of the canonical GT blast pulse is rather unwieldy, but can be evaluated from

(A80) $$g_{ℋ}\left(\hat{\tau }\right)=ℋ\left[g\left(\hat{\tau }\right)\right]=P\frac{1}{\pi }\int_{-\infty }^{\infty }\frac{g\left(x\right)}{t-x}dx$$

where the P in front of the integral denotes the Chaucy principal value. Multiple integration by parts over the interval of the GT pulse yields

(A81) $$g_{ℋ}\left(\hat{\tau }\right)=\frac{1}{\pi }\left[1+\left(1-\hat{\tau }\right)ln\left(-\hat{\tau }\right)-\left(1-\hat{\tau }\right)ln\left(1-\hat{\tau }\right)\right],\;0\leq \tau \leq 1\;g_{ℋ}\left(\hat{\tau }\right)=\frac{1}{6\pi }\frac{\left(a-1\right)}{6}\left[a\left(2a+5\right)-1+6{\hat{\tau }}^{2}-3\hat{\tau }\left(1+3a\right)\right]\;$$

(A82) $$+\;\frac{1}{6\pi }\left[\left(\hat{\tau }-1\right){\left(a-\hat{\tau }\right)}^{2}\right]\left[ln\left(a-\hat{\tau }\right)-ln\left(1-\hat{\tau }\right)\right],1<\tau \leq a=1+\sqrt{6}\;.$$

Since

(A83) $$\underset{x\to 0}{\lim}\;x\;ln\left(x\right)=0,\;\underset{x\to 0}{\lim}\;x^{2}\;ln\left(x\right)=0$$

the solutions are well behaved near the zero crossings. However, there are some issues in this solution. First, there are the two troublesome implicitly complex terms. The first is

(A84) $$ln\left(-\hat{\tau }\right)=ln\left(\hat{\tau }\right)+j\pi ,\;0\leq \tau \leq 1$$

where $ln\left(\hat{\tau }\right)$ tends to negative infinity at $\hat{\tau }=0$. The second tricky term is

(A85) $$ln\left(1-\hat{\tau }\right)=ln\left(\hat{\tau }-1\right)+j\pi ,\;1<\tau \leq 1+\sqrt{6}\;.$$

The complex terms are awkward; fortunately, multiplication and division by zero can be readily avoided numerically by adding the smallest floating point value (float epsilon) to arguments in logarithmic computations so it is possible to evaluate the real part of the solution. Another inconvenience is the discontinuity in $g_{ℋ}$ and its slope as $\hat{\tau }\to 1$. Rewriting the first term as

(A86) $$g_{ℋ}{\left(\hat{\tau }\right)}_{\hat{\tau }<1}=\frac{1}{\pi }\left[1+\left(1-\hat{\tau }\right)ln\left(\hat{\tau }\right)-\left(1-\hat{\tau }\right)ln\left(1-\hat{\tau }\right)\right]+j\left(1-\hat{\tau }\right),\;\hat{\tau }\to 1\;from\;below$$

(A87) $$g_{ℋ}\left(\hat{\tau }\to 1\right)=\frac{1}{\pi }\;.$$

Evaluating the second term yields

(A88) $$g_{ℋ}\left(\hat{\tau }=1\right)=\frac{1}{\pi }\frac{\sqrt{2}}{\sqrt{3}}=\frac{1}{\pi }\left[1-\frac{\sqrt{3}-\sqrt{2}}{\sqrt{3}}\right],\;\hat{\tau }\to 1\;from\;above.$$

These deficiencies are suboptimal, and not altogether surprising given that the waveform did not design integrability into the GT pulse [7]. Fortunately, these inadequacies are deemed computationally irrelevant by using the numerical convolution provided by the SciPy [25] signal.hilbert, which returns the analytic function for a real input waveform. The comparison between the synthetic theoretical analytic signals, the CWT reconstruction, and the numerical Hilbert transform are presented in the figures in the main text.
